# IL-17–producing follicular Th cells enhance plasma cell differentiation in lupus-prone mice

**DOI:** 10.1172/jci.insight.157332

**Published:** 2022-06-08

**Authors:** Vera Kim, Kyungwoo Lee, Hong Tian, Su Hwa Jang, Betty Diamond, Sun Jung Kim

**Affiliations:** 1 Center for Autoimmune, Musculoskeletal, and Hematopoietic Diseases, Institute of Molecular Medicine, The Feinstein Institutes for Medical Research, Manhasset, New York, USA.; 2 Department of Biomedical Science, Graduate School of Biomedical Sciences and Engineering, Hanyang University, Seoul, South Korea.; 3 Department of Molecular Medicine, Donald and Barbara Zucker School of Medicine, Hofstra University, Hempstead, New York, USA.

**Keywords:** Autoimmunity, Immunology, Autoimmune diseases, Cytokines, Lupus

## Abstract

Follicular Th (Tfh) cells are key CD4^+^ Th cells involved in regulating B cell differentiation in the germinal center. Mice with conditional KO (CKO) of B lymphocyte-induced maturation protein-1 (BLIMP-1) expression in CD11c^hi^ DCs (*Prdm1* CKO) spontaneously develop a lupus-like disease. We found an increase in the number of Tfh cells in secondary lymphoid organs in lupus-prone *Prdm1*-CKO mice. B cells stimulated with Tfh cells become Ab-secreting cells (ASCs); there was an increase in ASC differentiation mediated by Tfh cells from *Prdm1*-CKO mice compared with Tfh cells from control mice. This was mainly due to the increased expression of molecules within the IL-23/IL-17 pathway in Tfh cells from *Prdm1*-CKO mice. There was an increased frequency of IL-17A^+^ Tfh cells in *Prdm1*-CKO mice. These Tfh cells secrete IL-17A and an increased amount of IL-21. Furthermore, neutralizing IL-17A and IL-21 reduced ASC differentiation induced by Tfh cells. Lastly, we found the expression of IL-1β and IL-6 was increased in BLIMP-1–deficient DCs, which are key for Th17 induction. Altogether, the lack of BLIMP-1 expression in DCs induces not only an increased number of Tfh cells, but also functionally distinct Tfh cells that lead to increased ASC differentiation.

## Introduction

The proper generation of CD4^+^ Th cells is required for both protective immune responses to pathogens and immune tolerance against self-antigens. An imbalance within Th subsets and alteration in their function can lead to deleterious autoimmune diseases, including systemic lupus erythematosus (SLE), multiple sclerosis, and rheumatoid arthritis (RA) (1). Among the Th subsets, follicular Th (Tfh) cells are crucial for the development of humoral immune responses. Differentiation of Tfh cells is initiated by stimulation with cognate antigen-presenting DCs in secondary lymphoid organs. DCs provide costimulatory signals for Tfh differentiation by secretion of cytokines and cell surface expression of costimulatory molecules, such as CD86/80 and OX40 ligand (OX40-L) (2). Expression of several key molecules, including B cell lymphoma 6 (BCL6), C-X-C Motif Chemokine Receptor 5 (CXCR5), and programmed cell death protein 1 (PD-1), is induced in Tfh cells by the initial contact with DCs. Activated Tfh cells move to the B cell-T cell boundary and into the B cell follicle where they further mature to become germinal center (GC) Tfh cells. GC Tfh cells express higher levels of BCL6, CXCR5, and PD-1 and become inducible costimulatory molecule-positive (ICOS-1–positive). Both CXCR5 and PD-1 are important for positioning of Tfh cells within the B cell region where they help B cells during GC responses (3). The transcription factor, BCL6, is a master regulator of Tfh differentiation. Tfh cells, in the GC, express ICOS-1 and produce high levels of IL-21, which are important for proliferation/survival of B cells and differentiation of plasma cells (4). Precise control of the number of Tfh cells is important to produce optimal immune protection devoid of self-reactivity. Excess numbers of Tfh cells appear to allow survival of low-affinity self-reactive B cells, leading to a loss of immune tolerance (5). The number of circulating Tfh (cTfh) cells is positively correlated with disease activity in SLE, suggesting that controlling the number of Tfh cells may be significant for prevention or treatment of disease.

Heterogeneity is a distinct feature of Tfh cells. Since their initial identification, Tfh cells have been subgrouped into Tfh1, Tfh2, and Tfh17 depending on the coexpression of BCL6 and other lineage-determining transcription factors, T-BET, GATA3, and RORγt, respectively (6, 7). cTfh cells in humans are defined by the combination of these markers, chemokine receptors CXCR3, and C-C Motif Chemokine Receptor 6 (CCR6) (8), and their production of cytokines. Not surprisingly, in addition to the increased number of Tfh cells, functional differences within the Tfh cells have been identified in patients with SLE compared with healthy individuals. Skewed expansion of Tfh17 or Tfh2 was observed in various lupus mouse models (9, 10).

In previous studies, we showed that mice with a conditional deletion of *Prdm1* in DCs (*Prdm1* CKO) develop a lupus-like syndrome with an increased number of Tfh cells and an increased number of GC B cells (11). This model is of interest as B lymphocyte-induced maturation protein-1 (BLIMP-1) deficiency in DCs phenocopies a SLE *PRDM1* risk allele (12). In *Prdm1*-CKO mice, the number of Tfh cells was increased in the secondary lymphoid organs and their TCRβ repertoire was distinct from control mice (13), but the function of Tfh cells in these mice was not addressed. Here we showed that Tfh cells are not only expanded in number but were also functionally altered, leading to an increased B cell activation in *Prdm1*-CKO mice. The Tfh cells of *Prdm1*-CKO mice secrete higher amounts of IL-21 and IL-17, secondary to increased IL-1β production from BLIMP-1–deficient DCs and IL-6 as we previously showed. This alteration may explain the altered B cell activation, which leads to pathogenic autoantibodies in SLE.

## Results

### Increased B cell activation and plasmablast differentiation by Tfh cells from Prdm1-CKO mice.

**Figure 1:**
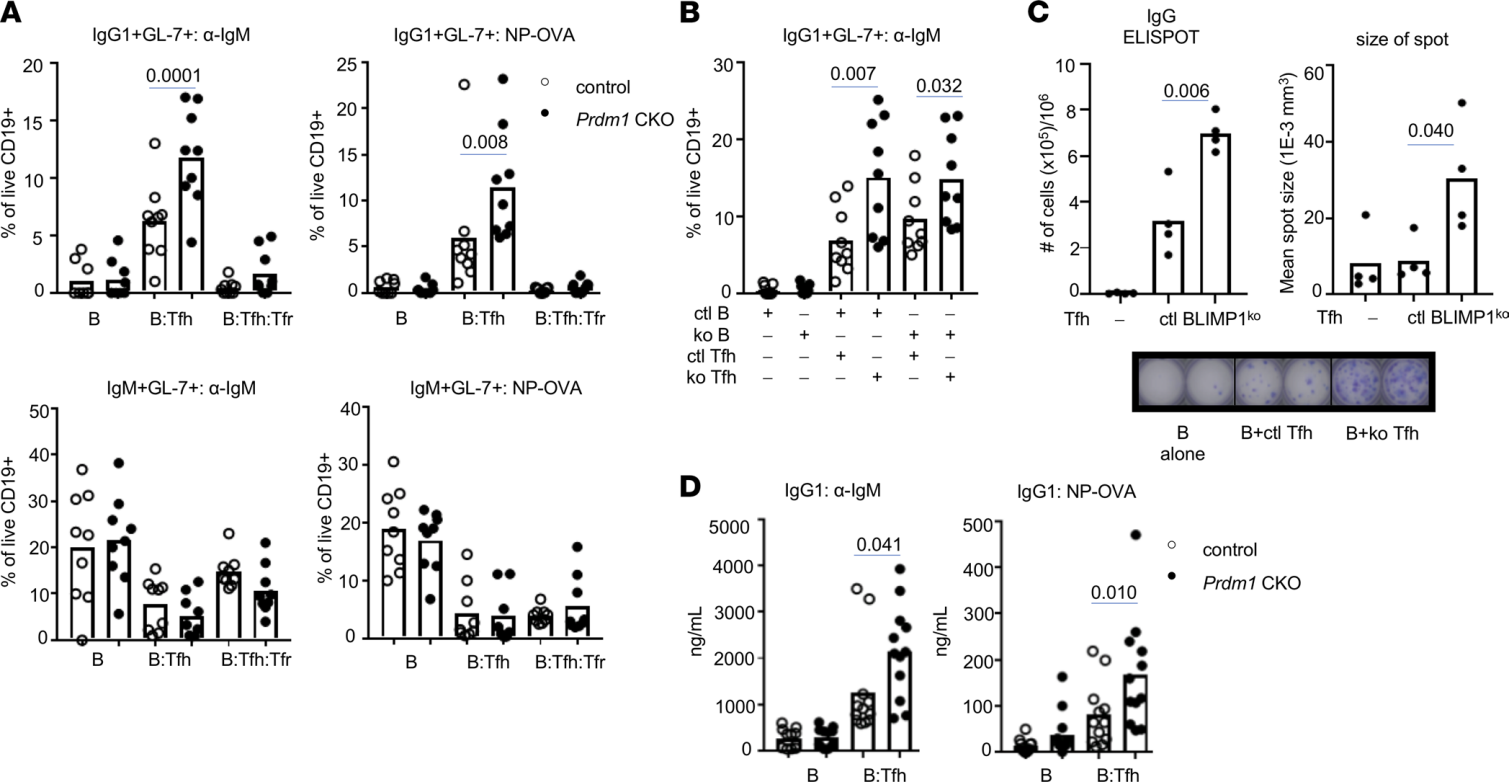
Increased IgG1^+^ PB differentiation by Tfh cells from *Prdm1-*CKO mice. FOXP3-GFP, *Prdm1*-CKO or FOXP3-GFP, and control mice (6- to 8-week-old females) were immunized with NP-CGG and live B cells (CD19^+^), Tfh (TCRβ^+^CD4^+^CXCR5^+^PD1^+^FOXP3^–^), and Tfr (TCRβ^+^CD4^+^CXCR5^+^PD1^+^FOXP3^+^) cells were isolated by cell sorter. (**A**) Isolated B cells were cultured alone, with Tfh, or with Tfh and Tfr in the presence of anti-IgM with anti-CD3 antibodies or NP-OVA with anti-CD3 antibodies as indicated in the figure (legend indicates the source of T cells; open circle is CTL mice and closed circle is *Prdm1*-CKO mice). After 7 days of culture, isotype-specific PB was identified by staining of GL7, IgG1 (upper graph), and IgM (bottom graph) (*n* = 9 for both CTL and KO). (**B**) IgG1 PB differentiation was performed with B cells from CTL mice or *Prdm1*-CKO mice (*n* = 9 for both CTL and KO). (**C**) Number of IgG1-secreting PB was quantified by ELISpot. A representative image of spots from 4 independent experiments (duplicates of each culture condition) and the graphs indicate the number of spots (on the left) and the mean spot size (on the right) (*n* = 4). (**D**) Secreted IgG1 in the supernatant was measured by ELISA. Each dot represents an individual mouse in **A**, **B**, and **D** or an individual experiment in **C**. Bar indicates the mean (*n* = 12) from 3 independent experiments. A 1-way ANOVA with Bonferroni correction was used for statistical analyses.

We have previously reported that there are increased Tfh cells in the secondary lymphoid organs of *Prdm1*-CKO mice compared with littermate control (CTL) mice. To test whether Tfh cells from *Prdm1*-CKO mice contribute to enhanced B cell activation, B cells were cocultured with Tfh cells from either CTL mice or *Prdm1*-CKO mice. After 7 days of coculture, activated B cells were identified by the expression of GL7 and intracellular Ig by flow cytometry as reported (14). B cells stimulated ex vivo through the B cell receptor (BCR) with either anti–IgM or 4-Hydroxy-3-nitrophenylacetyl-OVA (NP-OVA) require Tfh cells to become activated and undergo class-switching recombination (CSR). There was a higher percent of IgG1^+^ GL7^+^ B cells, but not IgM^+^ GL7^+^ B cells, when the Tfh cells in the coculture were from *Prdm1*-CKO mice than CTL mice (Figure 1A). We also measured IgG2^+^ or IgG3^+^ GL7^+^ B cells, but none of these were induced in the in vitro culture (data not shown). It is possible that enhanced Tfh cells and GC B cells resulted from fewer Tregs or their lack of suppressive function in *Prdm1*-CKO mice. There was a decrease in the percentage of Treg cells in the spleens of *Prdm1-*CKO mice but the number of Tregs was similar in the spleens of *Prdm1-*CKO and CTL mice (Supplemental Figure 1A; supplemental material available online with this article; https://doi.org/10.1172/jci.insight.157332DS1). We also compared the generation of follicular regulator T (Tfr) (CXCR5^+^PD1^+^FOXP3^+^) in NP-chicken gamma globulin–immunized (NP-CGG–immunized) mice. Tfr was induced by immunization in both groups of mice, and the number of Tfr cells was not different in the spleens of *Prdm1* CKO compared with the CTL mice (Supplemental Figure 1B). Tfr cells from both CTL and *Prdm1*-CKO mice strongly suppressed plasmablast (PB) differentiation (Figure 1A), suggesting that Tfr cells were functionally competent in *Prdm1-*CKO mice.

The increased induction of IgG1^+^ GL7^+^ B cells by Tfh cells from *Prdm1-*CKO mice occurred with B cells from either CTL or *Prdm1*-CKO mice, implying this is Tfh-cell intrinsic (Figure 1B). The IgG1^+^ GL7^+^ B cells secrete Ig and are PBs assessed by ELISpot and IgG1 in the supernatant by ELISA. Both experiments showed that there was an increased number of Ab-secreting cells (ASCs) and a higher level of IgG in the supernatant from B cells cultured with Tfh cells from *Prdm1*-CKO mice compared with B cells with Tfh cells from CTL mice (Figure 1, C and D). In addition to the increased number of ASCs, the size of the spots was significantly bigger when B cells were cultured with Tfh cells from *Prdm1-*CKO mice (Figure 1C). The increased IgG1 PB differentiation was also observed with Tfh from lymph nodes (LNs), suggesting the functional changes in Tfh are not restricted to the spleen (Supplemental Figure 2).

### Enhanced expression of genes in IL-23/IL-17 pathway in Tfh cells from Prdm1-CKO mice.

**Figure 2:**
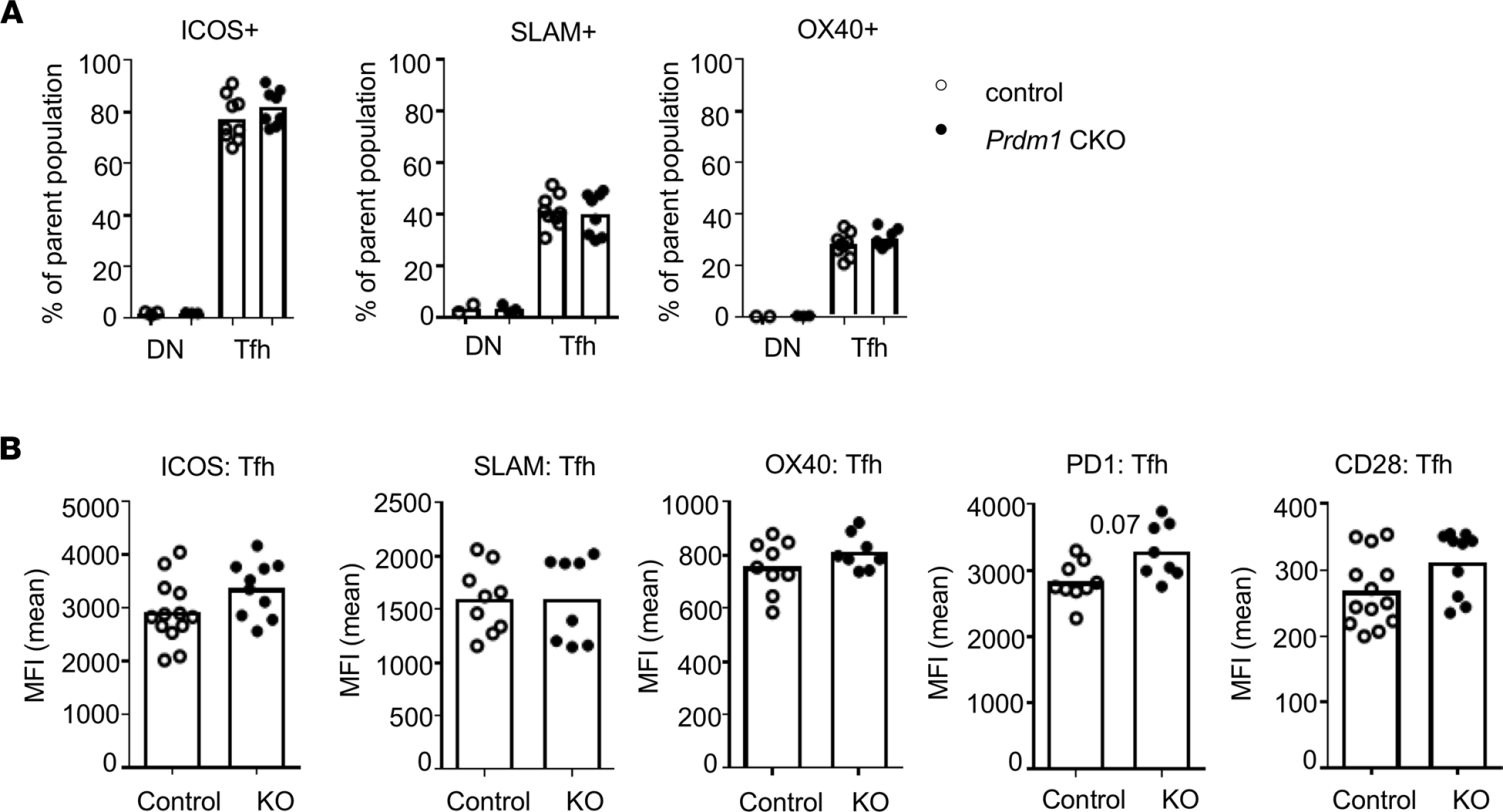
Expression of costimulatory molecules on Tfh cells. FOXP3-GFP *Prdm1*-CKO and CTL mice (6- to 8-week-old females) were immunized with NP-CGG and spleens were isolated after 7 days. Costimulatory molecules, ICOS1, SLAM, OX40, PD1, and CD28, were stained and the percent of positive cells in Tfh cells (CXCR5^+^PD1^+^FOXP3^–^) or in DN (CXCR5-PD1-) within CD4 T cells are presented in (**A**) and the MFI of expression level of each molecule of Tfh cells is presented in (**B**). An open circle represents control mice and the closed circle represents *Prdm1-*CKO mice. Each dot represents an individual mouse, and the bar graph indicates the mean (*n* = 13 for control and *n* = 10 for KO) from 3 independent experiments. An unpaired 2-tailed Student’s *t* test (Mann-Whitney) was used.

To understand how Tfh cells of *Prdm1*-CKO mice induce enhanced B cell activation, we evaluated the expression of costimulatory molecules. Engagement of costimulatory molecules, including CD28, ICOS, OX40L, signaling lymphocytic activation molecule (SLAM), and PD-1 between T and B cells is required for B cell activation. Expression of costimulatory molecules was assessed in Tfh cells from mice 7 days after immunization. Most of the costimulatory molecules are highly expressed in Tfh cells compared with CXCR5/PD-1 double negative CD4^+^ T cells, but there was no difference in the percent of Tfh cells positive for ICOS, SLAM, or OX40 (Figure 2A) or the intensity of expression of ICOS, PD1, or CD28 in Tfh cells (Figure 2B) between CTL and *Prdm1-*CKO mice.

**Figure 3:**
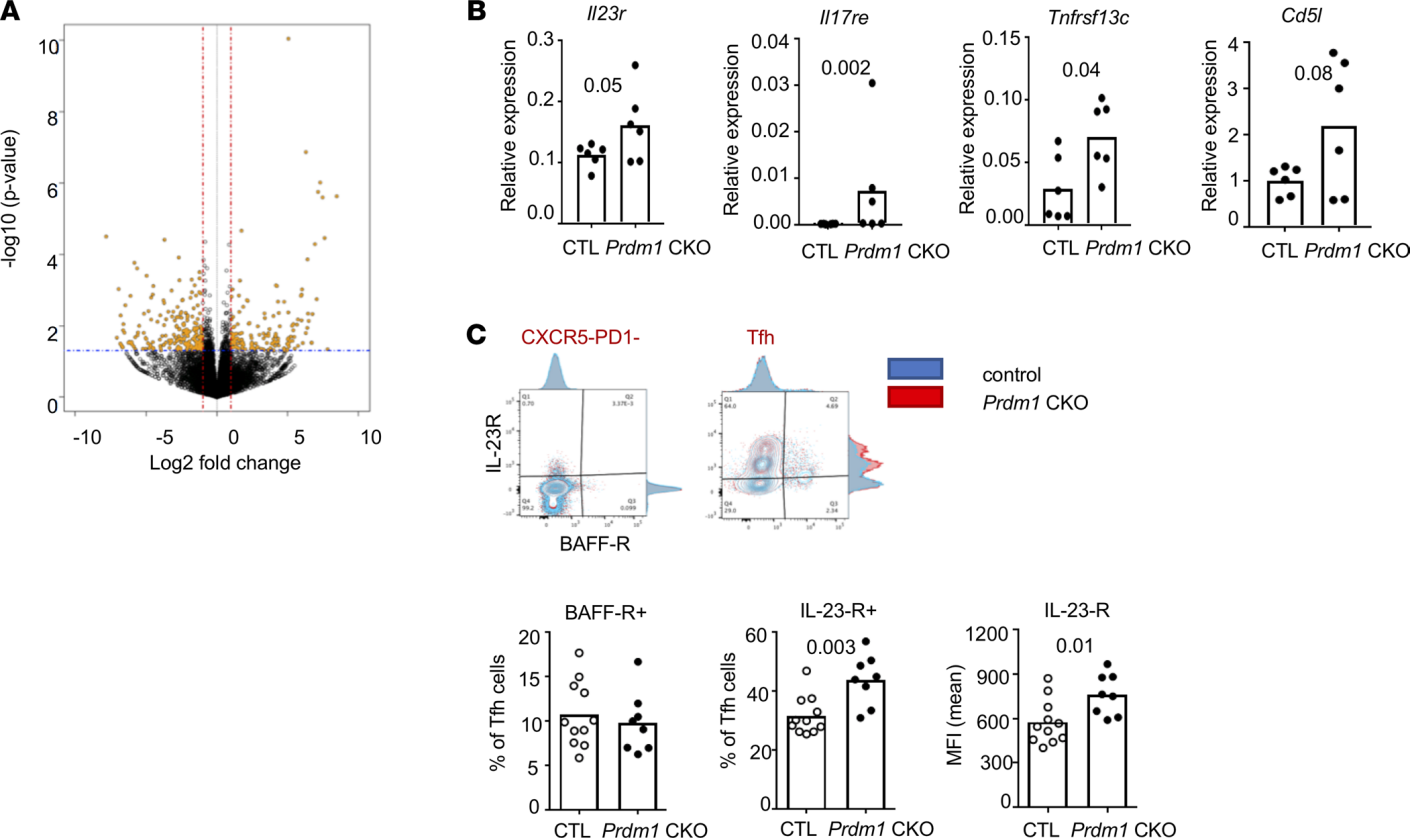
RNA-seq reveals an increased IL-17 pathway in Tfh cells from *Prdm1*-CKO mice. NP-CGG–immunized *Prdm1*-CKO and CTL mice were sacrificed at day 7 after immunization and transcriptome analysis were done by RNA-seq. (**A**) A volcano plot of differentially expressed genes was plotted. Positive fold change means gene expression is higher in *Prdm1*-CKO mice than control mice. To confirm the result in **A**, a separate cohort of mice was immunized and differential expression of genes in Tfh cells was measured by qPCR (**B**) (*n* = 6 for both CTL and KO). Protein expression of BAFF-R and IL-23R in CD4^+^ T cell subsets was measured by flow cytometry (**C**) (*n* = 11 for CTL and *n* = 8 for KO). A representative flow image of staining is on the top. Each dot represents an individual mouse, and the bar graph indicates the mean from 3 independent experiments. An unpaired 2-tailed Student’s *t* test (Mann-Whitney) was used.

Since we found that the expression of costimulatory molecules on Tfh cells was not significantly altered in *Prdm1*-CKO mice, we decided to perform a more global study of Tfh cells. Tfh cells were isolated from NP-immunized, age-matched female *Prdm1* and CTL-CKO mice, and RNA-seq was performed. Differential gene expression in Tfh cells of *Prdm1-*CKO and CTL mice is presented as a volcano plot in Figure 3A. There were 185 genes with increased expression (cutoff > 2-fold, *P* < 0.05) and 330 genes with decreased expression (cutoff < –0.5-fold, *P* < 0.05) in Tfh of *Prdm1*-CKO mice compared with CTL mice (Supplemental Tables 1 and 2). We focused on genes that are known to be involved in T cell differentiation and activation. We validated the increased expression of *Il23r*, *Il17re*, *Tnfrsf13c* (gene-encoding BAFF-R protein), and *Cd5l* in Tfh cells from *Prdm1-*CKO mice compared with Tfh cells from CTL mice (Figure 3B). Protein expression of IL-23R and BAFF-R was measured by flow cytometry. IL-23R expression on the surface of Tfh cells was positively detected on Tfh cells but not in CXCR5/PD1 double-negative CD4 T cells. IL-23R expression was higher in Tfh cells from *Prdm1-*CKO mice compared with CTL mice in both the percent of positive cells and the intensity of staining (Figure 3C).

### BLIMP-1–deficient DCs induced IL-17–producing Tfh cells by production of IL-1β and IL-6.

**Figure 4:**
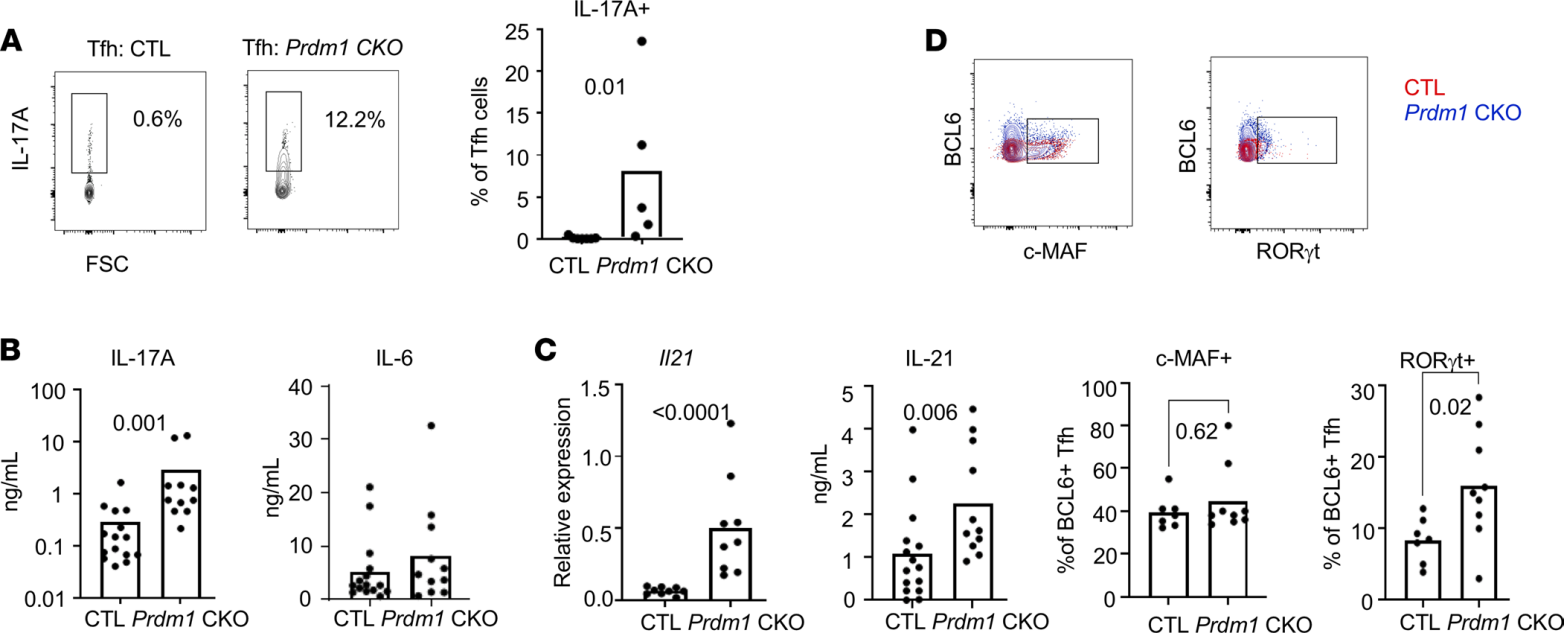
Increased IL-17– and IL-21–producing Tfh cells in *Prdm1-*CKO mice. Spleens were collected from NP-CGG–immunized *Prdm1*-CKO or control mice 7 days after immunization. (**A**) Intracellular IL-17A was measured in CD4^+^ Tfh cells by flow cytometry. Representative flow images are on the left panel and the graph is the quantitation of IL-17A^+^ cells within Tfh cells (*n* = 7 for CTL and *n* = 5 for KO). Three independent experiments were performed. (**B** and **C**) Isolated Tfh cells were cultured overnight, and the supernatant was collected for cytokine assay, the cells were lysed, and total RNA was isolated. IL-17, IL-6, and IL-21 levels were quantified by MSD and IL-21 mRNA was measured by qPCR. The bar represents the mean from 5 independent experiments and each dot represents an individual mouse (*n* = 15 for CTL and *n* = 11 for KO) (*n* = 9 for both CTL and KO, only for the 4C *Il21* qPCR experiment). (**D**) Intracellular staining of transcription factors (BCL6, c-MAF, and RORγt) was performed and measured within Tfh cells. Positive signal of BCL6 was obtained from Tfh cells and coexpression of c-MAF or RORγt was identified as in flow data (upper row). The percentage of c-MAF/BCL6 or RORγt/BCL6 was calculated from the total BCL6^+^ Tfh cells and the graph was made (bottom row). Each dot represents an individual animal and the bar represents the mean from 3 independent experiments (*n* = 7 for CTL and *n* = 9 for KO). An unpaired 2-tailed Student’s *t* test (Mann-Whitney) was used.

IL-23R signaling is important for Th17 cell differentiation (15). Although RNA-seq analysis did not show an increase in *IL-17* transcript in Tfh cells from *Prdm1-*CKO mice, increased expression of IL-23R and IL-17R prompted us to investigate IL-17 production in Tfh cells. IL-17 expression was assessed by intracellular staining in Tfh cells from NP-immunized mice. The frequency of IL-17 positive cells was dramatically increased in Tfh cells from *Prdm1-*CKO compared with CTL mice (Figure 4A). IL-17 secretion was measured by ELISA in ex vivo-stimulated Tfh cells from immunized mice. Consistent with the data from intracellular staining experiments, there was more IL-17A in the supernatant of Tfh cells from *Prdm1-*CKO mice (Figure 4B). We also measured other members of the IL-17 family (IL-17C, IL-17E/IL-25, IL-22) and IL-6 production since IL-6 is critical for Ab production and IL-6 expression was affected by BLIMP-1 in DCs in our previous studies (11). Tfh cells produce these cytokines, but no difference was observed between *Prdm1-*CKO and CTL mice (Figure 4B and Supplemental Figure 3).

IL-21 is a signature cytokine of Tfh cells and is the most potent cytokine for PB differentiation in both mice and humans (16, 17). We could detect IL-21 production by Tfh cells from both strains of mice. There was an increase in the IL-21 protein and mRNA expression (~ 2-fold) in Tfh cells from *Prdm1-*CKO mice compared with Tfh cells from CTL mice (Figure 4C). We also compared the expression of the transcription factor musculoaponeurotic fibrosarcoma (c-MAF) (positive regulator of *Il21* transcription) and RORγt (master transcription factor of Th17 cells) within the Tfh cells (CXCR5^+^PD1^+^BCL6^+^). We could detect c-MAF expression in Tfh cells, but there was no difference in Tfh cells between *Prdm1*-CKO and CTL mice. In contrast, we found increased RORγt expression in Tfh cells from *Prdm1*-CKO compared with CTL mice (Figure 4D).

**Figure 5:**
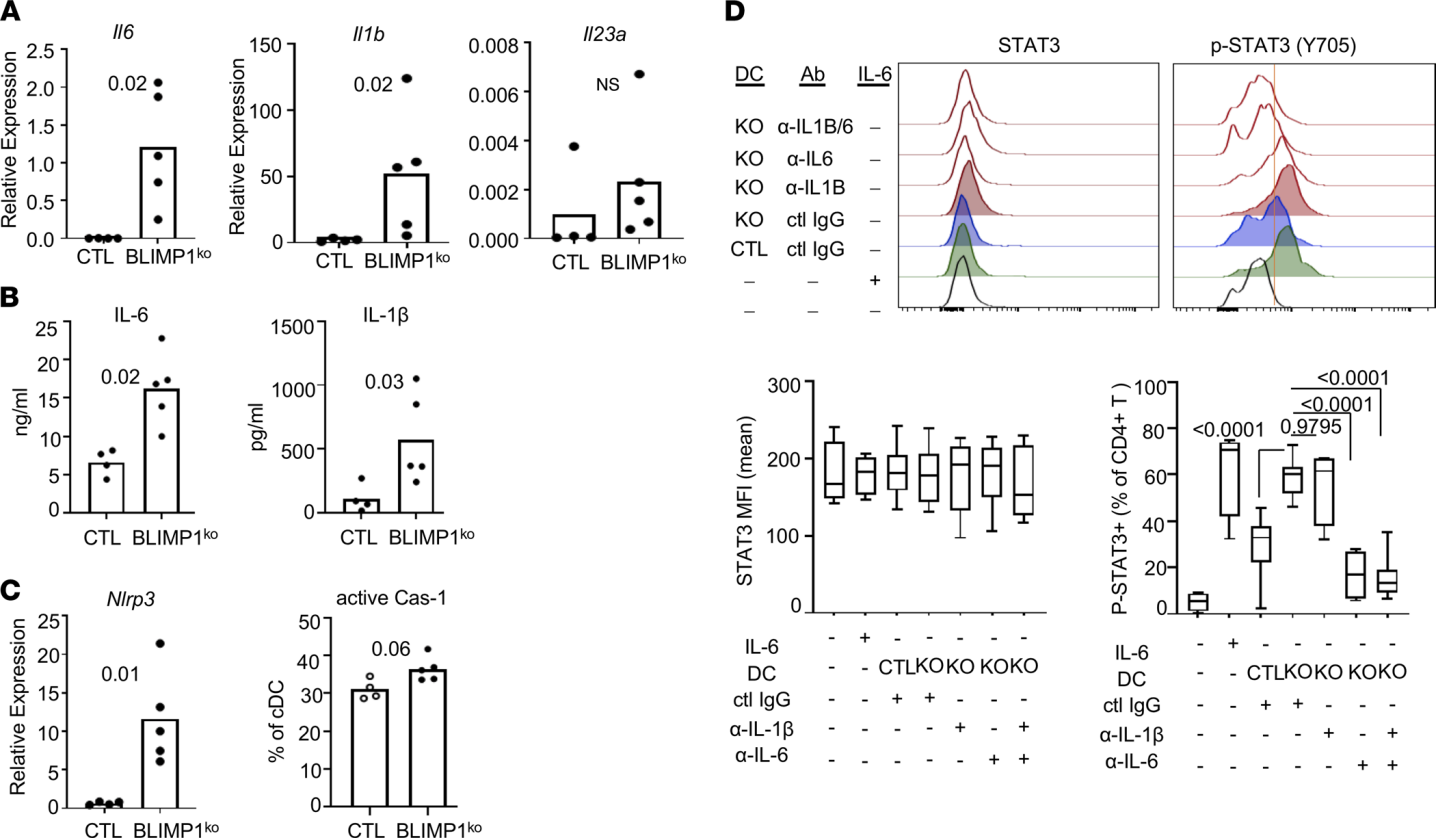
Increased activation of inflammasome and production of IL-1β and IL-6 in BLIMP-1–deficient DCs. *Prdm1-*CKO and control mice (6- to 8-week-old females) were immunized with NP-CGG, and spleens were collected at day 7 following immunization. cDCs (CD11c^hi^MHCII^hi^CD11b^+^) were purified by cell sorter and cultured for 4 hours, after which cells and supernatant were collected. (**A**) Transcript of cytokines were measured from the cells by qPCR, and (**B**) secreted cytokines in the supernatant were measured by MSD in 3 independent experiments (*n* = 4 for CTL and *n* = 5 or 6 for KO). (**C**) To measure the activation of the inflammasome pathway, transcripts of *Nlrp3* in cDCs were measured by qPCR and caspase-1 activity was measured by FAM-FLICA in cDCs. Each dot represents an individual mouse, and the bar represents the mean (*n* = 4 for CTL and *n* = 5 for KO) from 3 independent experiments. (**D**) STAT3 activation in naive T cells was measured. Naive CD4^+^ T cells and DCs were prepared, and T cells were stimulated with control DCs (CTL) or BLIMP-1–deficient DCs (KO) or rmIL-6 for 15 minutes. Neutralizing Abs of IL-6/IL-1β or control IgG were added during the stimulation in the indicated conditions. After the stimulation, cells were fixed and p-STAT3 and total STAT3 were measured by flow cytometry. Representative histogram images are on the upper row. A red line was drawn based on T alone without stimulation. Each dot represents an individual animal, and the bar represents the mean from 3 independent experiments (*n* = 12). An unpaired 2-tailed Student’s *t* test (Mann-Whitney) was used for **A–C** and 1-way ANOVA with Dunnett’s correction was used for **D**.

To understand the mechanism for the increase in IL-17 and IL-21 production in Tfh cells in *Prdm1*-CKO mice, we investigated cytokine production by DCs from immunized mice. We measured expression of *Il6*, *Il23a*, and *Il1b,* which are required for IL-21 and IL-17 production, respectively. There was increased expression of both *Il6* and *Il1b* in BLIMP-1–deficient DCs compared with CTL DCs. The expression of *Il23a* is very low in both groups of DCs, and we could observe only a trend toward an increase in IL-23 in BLIMP-1–deficient DCs (Figure 5A). We confirmed that the secretion of IL-6 and IL-1β by ELISA and both cytokines were higher in supernatant from BLIMP-1–deficient DCs than CTL DCs (Figure 5B). Secretion of IL-1β from the cells requires a cleavage of pro–IL-1β by active caspase-1 (18). Expression of *Nlrp3* and the level of active Caspase-1 is higher in BLIMP-1–deficient DCs supporting the increased secretion of IL-1β by BLIMP-1–deficient DCs (Figure 5C). Activation of the STAT3 pathway is required for Tfh differentiation and IL-21 expression in mice (19). BLIMP-1–deficient DCs induced enhanced STAT3 phosphorylation in naive CD4^+^ T cells compared with control DCs, and this could be prevented by anti–IL-6 Abs (Figure 5D). Although IL-1β secretion was higher by BLIMP-1–deficient DCs, IL-1β did not affect STAT3 activation in T cells as there was a minimal effect on STAT3 activation by the anti–IL-1β Ab (Figure 5D).

**Figure 6:**
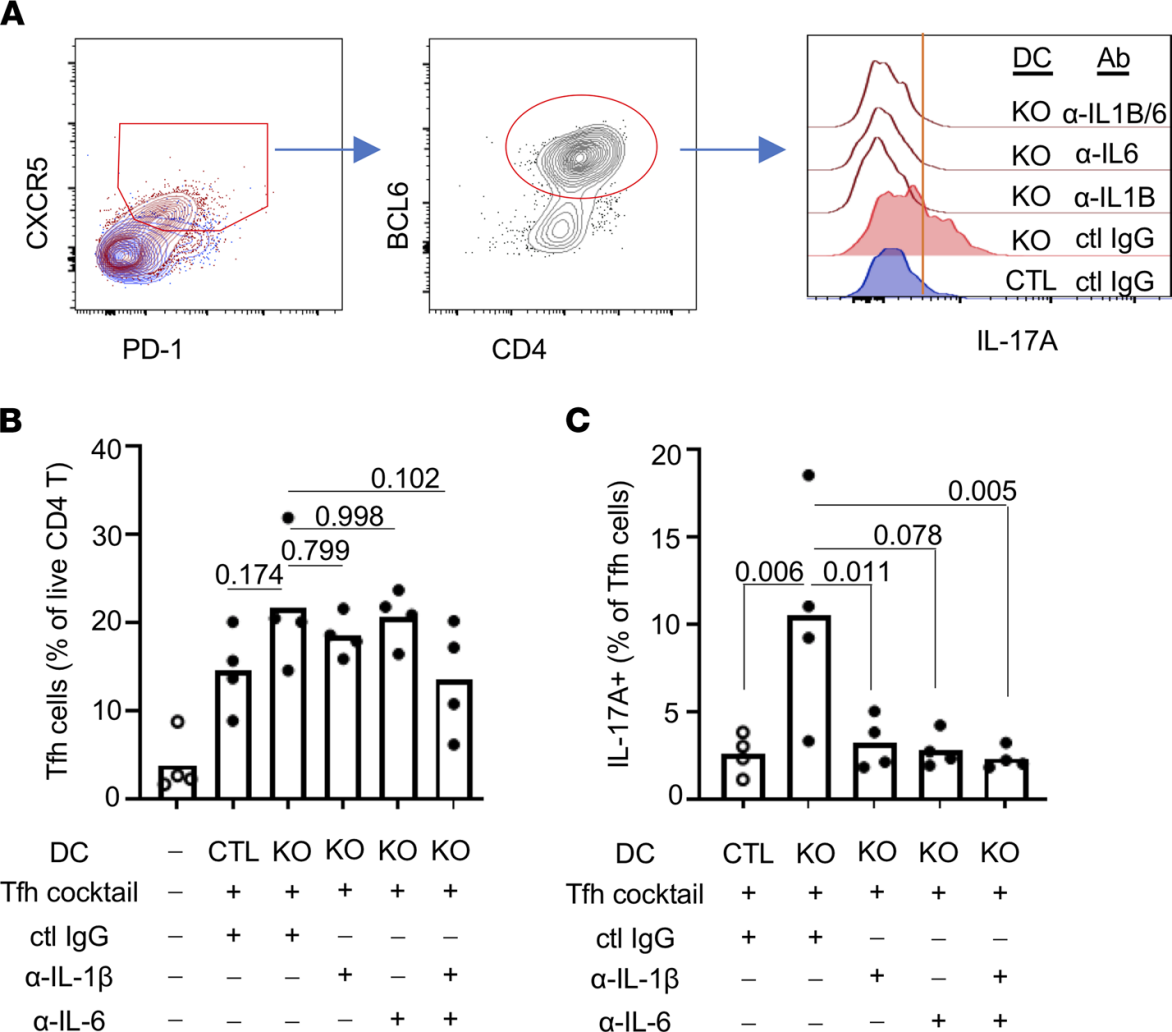
IL-6 and IL-1β are required for IL-17A production in Tfh cells. Spleens were collected from CTL or *Prdm1*-CKO mice on day 7 following NP-CGG immunization. DCs and B cells were cocultured with naive CD4^+^ T cells under Tfh differentiation condition. Neutralizing Ab of IL-6/IL-1β or control IgG was added during the stimulation in the indicated conditions. After 4 days in culture, cells were stimulated, fixed, and intracellular IL-17A was measured in CXCR5^+^PD1^+^CD4^+^BCL6^+^ Tfh cells by flow cytometry (**A**). Tfh cells within live CD4^+^ T cells (**B**) and IL-17A within Tfh cells (**C**) were measured by flow cytometry. Open circles represent CTL mice and the closed circles represent *Prdm1-*CKO mice. Each dot represents an individual mouse, and the bar graph represents the mean from 3 independent experiments (*n* = 4). An unpaired 2-tailed Student’s *t* test (Mann-Whitney) and 1-way ANOVA with Dunnett’s correction were used.

Next, we investigated whether IL-1β and IL-6 contribute to differentiation of IL-17^+^ Tfh cells using the in vitro Tfh differentiation assay. DCs and B cells were isolated from NP-CGG–immunized CTL or *Prdm1*-CKO mice. Naive CD4^+^ T cells were isolated from CTL mice and cocultured with DCs under Tfh differentiation conditions (11). Since IL-6 is required for Tfh differentiation, we added a low dose of anti–IL-6 Ab to prevent complete blocking of Tfh differentiation. There was a similar level of Tfh differentiation (CXCR5^+^PD1^+^BCL6^+^ expression), but this was unaffected by neutralizing Abs (Figure 6, A and B). However, the percentage of IL-17A–expressing Tfh cells was significantly decreased when the culture included either anti–IL-6 or anti–IL-1β Ab (Figure 6, A and C). These data suggest that an increased secretion of IL-6 and IL-1β from BLIMP-1–deficient DCs contributes to enhanced IL-17 and IL-21 in Tfh cells.

### Increased production of IL-17 and IL-21 from Tfh cells are responsible for enhanced B cell differentiation.

**Figure 7:**
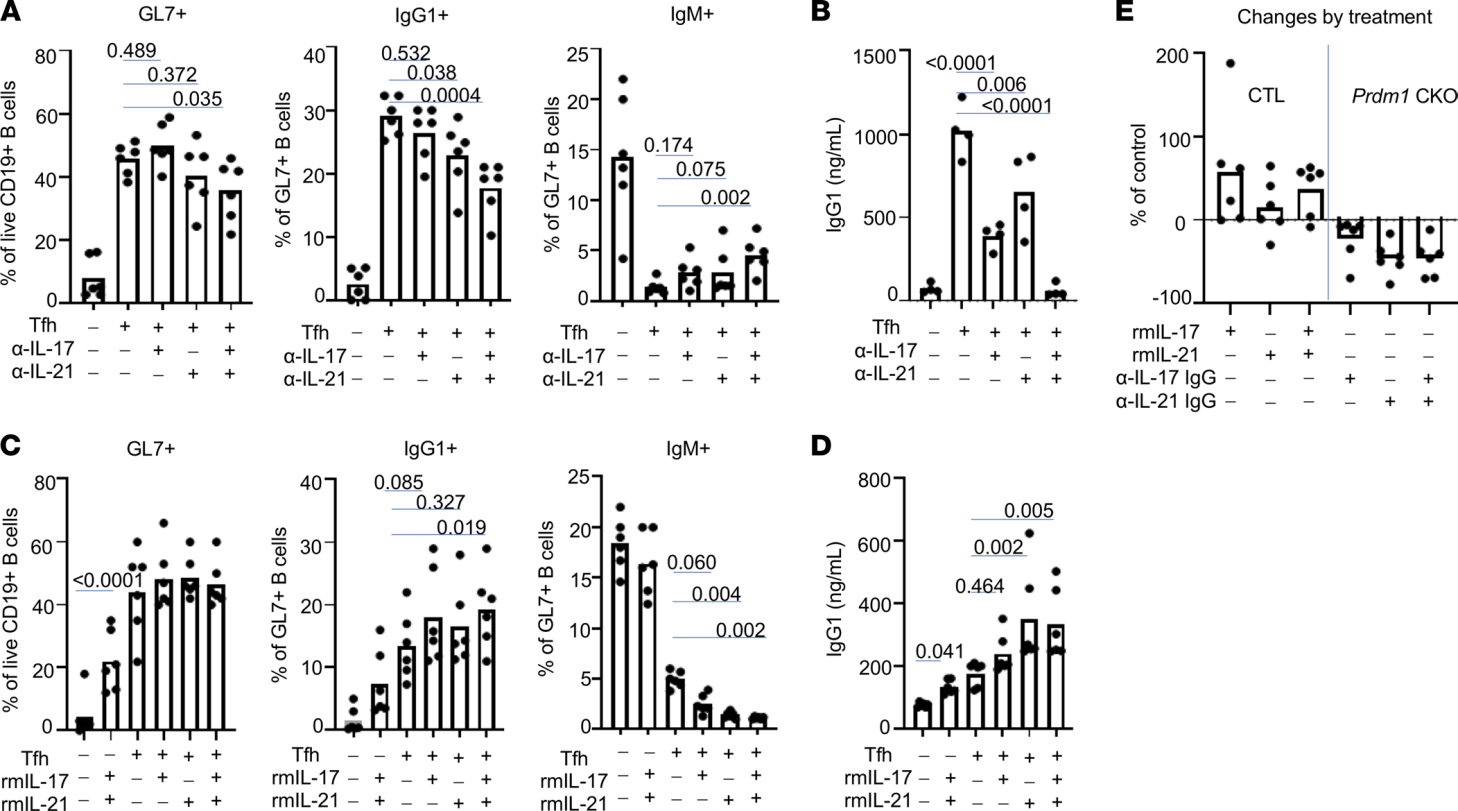
IL-17 and IL-21 from *Prdm1*-CKO Tfh cells are responsible for the increased PB differentiation. (**A**) B cells and Tfh were isolated from NP-CGG–immunized *Prdm1*-CKO mice, and they were cocultured in the presence of anti–IL-17 (10 μg/mL), anti–IL-21 (1 μg/mL) neutralizing Abs, or control IgG (10 μg/mL) as indicated in the figure. Proliferating B cells and IgG1 PB or IgM PB were identified by staining with GL7 and intracellular IgG1 and IgM within FVD-negative live B cells (*n* = 6, 3 independent experiments). (**B**) Supernatant from the **A** experiment was collected and IgG1 was quantified by ELISA (*n* = 4, 3 independent experiments). (**C**) B cells and Tfh were isolated from NP-CGG–immunized control mice, and they were cocultured in the presence of rmIL-17 (1 ng/mL) or rmIL-21 (1 ng/mL). Proliferating B cells, IgG1 PB, and IgM PB were identified by staining with GL7 and intracellular IgG1 and IgM (*n* = 6, 3 independent experiments). (**D**) Supernatant from the **C** experiment was collected and IgG1 was measured by ELISA. (**E**) The difference in IgG1 PB was calculated as follow: for CTL Tfh (first 3 columns), (IgG1 PB from rm cytokine-treated groups IgG1 PB of no exogenous cytokine group)/IgG1 PB of no exogenous cytokine group x 100; for *Prdm1*-CKO Tfh (last 3 columns), and (IgG1 PB from neutralizing IgG–treated group IgG1 PB of ctl-IgG–treated group)/IgG1 PB of ctl-IgG–treated group x 100. Each dot represents an individual mouse, and the bar graph represents the mean (*n* = 6, 3 independent experiments). A 1-way ANOVA with Dunnett’s correction was used.

To test whether the increased IL-17 and IL-21 from Tfh cells are responsible for increased PB differentiation, we blocked IL-17 or IL-21 during the in vitro coculture of B cells and Tfh from *Prdm1*-CKO mice. Neutralizing Abs against either IL-17A or IL-21 showed little impact on induction of GL7^+^-proliferating B cells. However, blocking of these cytokines significantly suppressed IgG1^+^ GL7^+^ B cells (Figure 7A) and secretion of IgG1 (Figure 7B). The decreased IgG1^+^ B cells is presumably due to the blocking of CSR since the percentage of IgM^+^ GL7^+^ B cells was higher in cultures with anti–IL-17/IL-21 than control IgG (Figure 7A). Interestingly, the combination of anti–IL-17 and anti–IL-21 showed synergistic suppression in IgG1 secretion and modest inhibition of B cell activation as well. We also added recombinant IL-17 or IL-21 into the coculture of B cells and Tfh cells from control mice. Exogenous cytokines increased activation of B cells in cultures of B cells alone, but not in B cells with Tfh cells. IL-17 or IL-21 decreased the percentage of IgM^+^ GL7^+^ B cells and trends toward an increase in the percentage of IgG1^+^GL7^+^ cells, though it was not statistically significant (Figure 7C). The increase was confirmed by an increased secretion of IgG1 by exogenous IL-21 or IL-21/IL-17, but not IL-17 alone, from B cells and B cells with CTL Tfh (Figure 7D). The change of IgG1 PB by treatment of recombinant cytokines or neutralizing antibody was calculated (Figure 7E). These data suggest that increased secretion of IL-17 or IL-21 from Tfh cells is responsible at least partly for IgG1 PB differentiation.

## Discussion

In this study, we demonstrated that Tfh cells in lupus-prone *Prdm1*-CKO mice are not only increased in number but are also different functionally from Tfh cells in CTL mice. There was increased production of IL-21 and IL-17A in Tfh cells in *Prdm1-*CKO mice. Tfh cells from *Prdm1-*CKO mice showed stronger B cell activation and induced more IgG1 PB differentiation in vitro. Increased IL-21 and IL-17 are important for PB differentiation by Tfh cells since blockade of these cytokines suppressed differentiation into IgG1^+^ PB without changing B cell proliferation. Compared with CTL DCs, BLIMP-1–deficient DCs produced higher amounts of IL-1β and IL-6 after immunization, and these cytokines may play a critical role in differentiation of IL-17/IL-21–producing Tfh cells in *Prdm1-*CKO mice.

It has been accepted that an expanded Tfh cell number can break B cell tolerance and an expansion of Tfh cells has been observed in various mouse models of lupus (2, 20). The increased Tfh cells in the circulation are known to be positively correlated with disease activity or the level of anti-dsDNA Abs in patients with SLE (21). Our previous study found an expansion of Tfh cells contributing an expansion of GC B cells and development of autoreactive Abs in female *Prdm1*-CKO mice. The random nature of somatic hypermutation permits B cells to acquire autoreactivity in the GCs and an excessive number or overactivation of Tfh cells can corrupt negative selection, allowing survival of autoreactive B cells. Therefore, the expansion of Tfh cells and GC B cells should be tightly regulated by Tregs (Tfr) and failure of Tfr can lead to an aberrant GC phenotype (14). Therefore, we assessed the regulatory T cells in lupus-prone *Prdm1*-CKO mice. The number of Treg cells is equivalent to the number in CTL mice, and, more importantly, they have intact suppressive function, suggesting that the expansion of Tfh cells and B cell activation did not result from the lack of Treg or from inadequate Treg function in *Prdm1*-CKO mice. Instead, the difference was due to a functional difference of Tfh cells since the same number of Tfh cells from either group of mice was added to B cell cultures. There was no difference in the expression of costimulatory molecules in Tfh cells between CTL and *Prdm1-*CKO mice. However, Tfh cells in *Prdm1-*CKO mice have a heightened Th17 pathway and secrete an increased level of IL-21. It is known that IL-21 and IL-17 work positively for plasma cell differentiation (22, 23) and the blocking of these cytokines effectively decreased the number of PBs and the level of secreted IgG1 during our in vitro culture with Tfh cells from *Prdm1*-CKO mice. Similarly, exogenous recombinant IL-17 or IL-21 increased the level of secreted IgG1 in Tfh and B cocultures but did not increase the number of PBs. This suggests that there are additional molecular changes for PB survival or PB proliferation present in Tfh cells from *Prdm1*-CKO mice.

Our finding of increased IL-17^+^ Tfh cells (potentially a counterpart of Tfh17 in humans) in *Prdm1*-CKO mice is interesting, since this phenocopies patients with autoimmune diseases (2). Patients with juvenile dermatomyositis displayed a decrease in the percent of Tfh1 subsets and an increase in the percent of Tfh2 and Tfh17 subsets among Tfh cells in the blood (8). In patients with SLE, an increased activation status of circulating Tfh cells has been observed (21). A selective increase in Tfh17 subsets has been positively correlated with disease activity in SLE (21). Moreover, these alterations in Tfh subsets are a rather stable phenotype over years in patients with SLE (24). These studies underscore that functional changes of Tfh cells are important for pathogenesis of disease. Tfh17 cells produce IL-17 in addition to IL-21 in both mice and humans (25). Tfh17 cells appear to share many features with Th17 cells, but the regulation of their development and function are not clearly understood. BCL6 and c-MAF are required for expression of key molecules, including IL-21 and ICOS, but whether they directly regulate IL-17 is not clear. Neither is the role of c-MAF in Tfh cells in Prdm1-CKO mice clear, since we did not find a difference in the level of expression. Instead, we found the increased expression of RORγt in Tfh cells from Prdm1-CKO mice, suggesting that RORγt may be responsible for differentiation of IL-17^+^ Tfh cells similar to Th17 cells. Th17 cell differentiation depends on IL-6 plus TGF-β, TGF-β plus IL-21, IL-1β plus IL-6, or IL-1β plus IL-23 (26–28); whether the same combination induces Tfh17 differentiation is not known. In our mouse model, we did not find a difference in IL-23 production from BLIMP-1–deficient DCs although there was an increased expression of IL-23R in Tfh cells. Instead, BLIMP-1–deficient DCs secrete more IL-6 and IL-1β. These cytokines are required for production of IL-17 by Tfh17 as neutralizing Abs significantly inhibit this process.

The increased production of IL-1β from BLIMP-1–deficient DCs is potentially important. In previous studies, we observed an increase in IL-1β production from colonic DCs in acute colitis-induced *Prdm1*-CKO mice (29). In addition to Th17 cell differentiation, IL-1β has shown to activate the production of IL-4 and IL-21 by Tfh cells in vitro (30) which will augment B cell activation. IL-1β induced a strong proliferation of Tfh cells, and this may contribute to the increased proportion of Tfh cells in *Prdm1*-CKO mice. We will need to confirm whether any of the functional changes in Tfh cells depend on IL-1β from BLIMP-1–deficient DCs.

The immunomodulatory function of IL-17 on B cells and autoantibody production has been shown as well. IL-17 enhances T-B interactions leading to GC development (9) and increases the longevity of long-lived plasma cells (22). Adoptive transfer of Th17 cells and PBMCs from patients with SLE into lymphocyte-deficient recipient mice leads to autoantibody production (31). IL-17 induces CSR to IgG1 and IgG2b (22), which are often high-affinity pathogenic Abs. Although we did not observe IgG2b class switching in our in vitro study, we observed a significant increase in IgG2 anti-dsDNA Abs development in vivo in our previous report (11). We also observed that blocking IL-17 effectively suppressed both PB differentiation and Ab secretion. However, the addition of IL-17 and IL-21 increased Ab secretion but not the number of class-switched PBs. These data suggest that there are other molecules from Tfh cells that cooperate with IL-17 for B cell activation.

The importance of Th17 cells and IL-17–producing T cells in disease progression and pathogenic roles has been observed. In mice, tissue infiltrating IL-17–producing cells have been identified in the kidney, skin, and secondary lymphoid organs in Murphy Roths Large/*lpr* (MRL/*lpr*) and B6-by-D2 (BDX2) mice model of lupus (32). In patients with SLE, there are high serum levels of IL-17 and an increased number of Th17 cells (33–35). Moreover, increased IL-17 levels were shown to be associated with poor histopathological outcomes after immunosuppressive treatment in patients with lupus nephritis (36). Indeed, high levels of IL-17 and related proinflammatory cytokines are detected in kidneys from patients with lupus nephritis (33). In line with these observations, IL-17^+^ T cells, including Th17 and CD4-CD8–double-negative (CD4-CD8–DN) T cells, have been found in kidneys from patients with SLE (33, 37). Therefore, a greater understanding of how Th17 cells and IL-17 are involved in the pathogenesis of SLE will help determine whether IL-17 is a promising therapeutic target. Novel biologic agent targeting directed toward IL-17 (secukinumab, ixekizumab, bimekizumab) and IL-17R (brodalumab) are approved for the treatment of psoriasis in the United States (38). A phase III, randomized, double-blind, placebo-controlled, 2-year study to evaluate the efficacy and safety of secukinumab in combination with standard therapy in patients with SLE with active nephritis (NCT04181762) is currently ongoing. Our findings show the importance of IL-17 and support the translation to clinical studies.

In summary, this study demonstrates a causal relationship between DC-driven functional changes in Tfh cells and B cell differentiation. This link substantiates the original hypothesis that changes in Tfh function as well as Tfh number are important for SLE.

## Methods

### Animals.

*Prdm1*-CKO (*Prdm1^fl/fl^*; *CD11c^–^CRE^+^*) mice and their CTL mice (*Prdm1^fl/fl^; CD11c^–^CRE^–^* and *Prdm1^+/+^*; *CD11c^–^CRE^+^*) were generated as reported in a previous study (11) by a stable breeder at the specific pathogen-free (SPF) animal facility at the Feinstein Institutes for Medical Research (FIMR). Foxp3-GFP *Prdm1-*CKO mice were generated by the crossmating of B6.Cg-Foxp3-GFP (The Jackson Laboratory, 006772) with *Prdm1-*CKO mice and housed at the FIMR animal facility.

Female 6- to 8-week-old mice were immunized with 100 μg NP-CGG (Biosearch Technologies,) in CFA either by i.p. or s.c.

### In vitro PB differentiation.

The in vitro PB differentiation protocol of the previous study was followed with slight modifications (14). Mice were immunized with NP-CGG either by s.c. or i.p. On day 7 following immunization, B cells ( $ $^+^), Tfh (TCRβ^+^CD4^+^CXCR5^+^PD1^+^FOXP3^–^), and Tfr (TCRβ^+^CD4^+^CXCR5^+^PD1^+^FOXP3^+^) were isolated by cell sorter, FACSAria, from spleens or LNs. A total of 5 × 10^4^ of B cells were cultured with or without 3 × 10^4^ Tfh alone or with 1.5 × 10^4^ Tfr. To stimulate T and B cells, anti-CD3 (2 μg/mL) and anti-IgM F(ab’)_2_ (5 μg/mL) were included in the culture medium. For the activation of antigen-specific T and B cells, NP-OVA (20 μg/mL) was added instead of anti-IgM. Cells were cultured for 6 days and PB differentiation was investigated by GL-7^+^ intracellular Ig (IgG1, IgM, IgG2, and IgG3) in the live B cells.

For certain experiments, 10 μg/mL of anti–IL-17–neutralizing Abs (Invitrogen, model 1402nAF); 1 μg/mL of anti–IL-21 (Invitrogen, model FFA21); 10 μg/mL mouse IgG1 kappa (Invitrogen, model P3.6.2.8.1); and 1 ng/mL of recombinant mouse (rm) IL-17 (R&D Systems) or 1 ng/mL of rmIL-21 (Peprotech) were added at day 0 of coculture.

### ELISpot assay.

A 96 well plate (Thermo Scientific, Immulon 2 HB) was coated with goat anti–mouse IgG1 Abs (10 μg/mL) (Southern Biotech, model 15H6) in PBS. The next day, the plate was washed with sterile PBS 3 times in hood and blocked with culture medium for 90 minutes at 37°C. Cells were counted and prepared in culture medium. Each sample was plated as a duplicate and 2-fold serial dilution to prevent saturation. Cells were incubated at 37°C in a CO_2_ incubator overnight. The next day, the plate was washed with wash buffer (PBS with 0.05% Tween 20) 5 times and incubated with polyclonal goat anti–mouse IgG-biotin (2 μg/mL) (Southern Biotech) in culture medium for 2 hours at 37°C. The plate was washed after the incubation 5 times with wash buffer and streptavidin-alkaline phosphatase (streptavidin-AP) (1:1000 dilution in medium) (Southern Biotech, catalog 7100-04) was incubated for 1 hour at 37°C. The plate was then washed 5 times with wash buffer and substrate (5-BCIP) in AMP buffer (0.75 mM MgCl2, 9.58% 2-amino-methyl-1-propanol, 0.01% Triton X, pH 10.25) and developed at room temperature for 1–2 hours until the spots became visible. The plate was rinsed with distilled water to stop the development and dry the plate. Spot counting and spot image were analyzed at Cellular Technology.

### Isolation of Tfh and RNA-seq.

Age-matched control and *Prdm1*-CKO female mice were sacrificed, and spleens were collected for Tfh isolation. Live CD4^+^ Tfh cells (FVD^–^TCRβ^+^CD4^+^CXCR5^+^PD1^+^) was sorted on the FACSAria (BD Bioscience), snap-frozen in liquid nitrogen, and kept at –80°C until sequencing.

RNA was purified by RNeasy micro kit (QIAGEN), and the quantity and quality of purified RNA was confirmed by Nanodrop 2000 (Thermo Scientific) and Agilent 2100 Bioanalyzer. RNA samples that had a higher RNA integrity number than 7 were further processed for library prep and sequencing. Library preparation and sequencing were performed at the Broad Institute by the Smart-seq2 method (39). Briefly, 50 ng of high-quality RNA was used for library construction and sequenced on an Illumina NextSeq500. A High-Output Kit was used to generate 2 x 25 bp reads (dual-index reads). Sequencing data were processed and aligned utilizing mm10 (University of California, Santa Cruz [UCSC]) and the corresponding GENCODE mouse genome (GRCm38) version 9 (Ensembl 84) annotation. FeatureCount (v1.4.6) was performed to convert the raw count files from aligned BAM files. Differential gene expression (DGE) analysis was performed utilizing DESeq2 with absolute values of log_2_ (fold change) greater than 1.5 as cutoffs to detect DGE. The RNA-Seq raw FASTQ files were deposited in the NCBI’s Gene Expression Omnibus database (GEO GSE201242).

### RNA isolation for qPCR.

Total RNA was isolated by RNeasy mini kit (QIAGEN) for RNA-seq and by Direct-zol RNA Micro Prep (Zymo Research) for quantitative PCR (qPCR). To avoid genomic DNA contamination, in-column DNA digestion was performed for all the RNA preparation per manufacturer’s protocol. The quantity of RNA was measured by nanodrop and 200–500 ng of total RNA was used for cDNA synthesis by iScript cDNA synthesis kit (Bio-Rad). This was followed by real-time quantification using qPCR Master Mix (Invitrogen) by gene-specific primers purchased from Taqman (Invitrogen) by Light Cycler 480 II (Roche): Mm00519942_m1 (*Il23r*); Mm01189488_m1 (*Il17re*); Mm00462151_m1 (*Cd109*); Mm00840578_g1 (*Tnfrsf13c*); Mm00437567_m1 (*Cd5l*); Mm00517640_m1 (*Il21*); Mm00446190_m1 (*Il6*); Mm00518984_m1 (*Il23a*); Mm00434228_m1 (*Il1b*); Mm00840904_m1 (*Nprl3*); Mm00839502_m1 (*Polr2a*); and Mm07297497_g1 (*Rplp1*). The difference in Ct values for expression for each gene was calculated by normalization to the level of housekeeping genes *Polr2a* and *Rplp1*.

### Phospho-STAT3 in T cells.

Naive CD4^+^ T cells were isolated and rested in complete media with a reduced serum (5% FCS) for 3 hours. DCs were purified by cell sorter from spleens of day 7 NP-CGG–immunized control or *Prdm1-*CKO female mice. Rested T cells were stimulated with DCs or 100 ng of rmIL-6 (positive control) for 15 minutes in an incubator at 37°C, then the stimulation was stopped by adding 20 volumes of Lyse/Fix Buffer (BD Pharmingen) for 10 minutes at 37°C. Cells were washed and surface staining of CD3 and intracellular staining of STAT3 and phospho-STAT3 were performed.

### In vitro Tfh differentiation.

In vitro Tfh differentiation was performed as described in a previous report (11) with slight modifications. DCs and B cells were isolated from spleens of 6- to 8-week-old control or Prdm1-CKO female mice 7 days after immunization with NP-CGG in CFA. Naive CD4^+^ T cells were isolated from spleens of CTL mice by EasySep isolation kit and cocultured with B cells and DCs at a 5:5:1 ratio, respectively, in the presence of Tfh differentiation cocktails (10 μg/mL of each anti–IL-4 (eBioscience, model 11B11), anti–IFN-γ (eBioscience, model XMF1.2), anti–TGF-β (R&D Systems, model 1D11), recombinant murine IL-6 (30 ng/mL), and IL-21 (50 ng/mL). To stimulate T cells, 2.5 μg/mL of anti-CD3 Ab (BD Pharmingen, model 145-2C11) was precoated on the plate or NP-OVA (10 μg/mL) was added. Neutralizing Abs against IL-1β (10 μg/mL; eBioscience, model B122), IL-6 (1 μg/mL; eBioscience, model MP5-20F3), or control IgG (10 μg/mL; eBioscience, model eBRG1) was added during the coculture. After 4 days of culture, Tfh cells were identified by expression of CXCR5, PD-1, and BCL6 in FVD-negative live CD4^+^ T cells. To measure the IL-17A expression, cells were stimulated with PMA (100 ng/mL), ionomycine (1 μg/mL), and brefeldin A (BFA) (20 μg/mL) for 5 hours on the day of assay. Intracellular staining for IL-17A and BCL6 was performed using True Nuclear staining kit (BioLegend) per manufacturer’s protocol.

### Flow cytometry analysis and Abs for flow cytometry.

Single cell suspensions (~ 2 × 10^6^ cells) were prepared and washed with cell staining buffer (2% FBS, 1 mM EDTA in PBS). Optimal concentration of each Ab was determined and Ab cocktail solution (including FVD to exclude dead cells) (eBioscience) was prepared in staining buffer. Cells were incubated with Ab cocktail solution for 15 minutes on ice, protected from direct light. After incubation, cells were washed twice with staining buffer. Intracellular staining was performed after completion of surface/FVD staining. Intracellular staining was performed with FOXP3 staining buffer kit (eBioscience) per the manufacturer’s protocol. To stain the intracellular cytokines, cells were stimulated with PMA (100 ng/mL; Sigma-Aldrich), ionomycin (1 μg/mL; Sigma-Aldrich), and BFA (20 μg/mL; BioLegend) for 6 hours before staining. Cells were acquired using a Fortessa flow cytometer (Beckman) and analyzed by FlowJo software (Tree Star).

The following Abs were purchased from BioLegend: anti-TCRβ (H57-597); anti-CD8 (53-6.7); anti-ICOS (C398.4A); anti–IL-23R (12B2B64),; anti-CD28 (37.51); anti–BAFF-R (7H22-E16); anti-SLAM (TC15-12F12.2); and anti-OX40 (OX-86). The following Abs were purchased from eBioscience: anti-CD4 (GK1.5); PD1 (J43); anti–IL-17A (eBio1787); anti–c-Maf (Sym0F1); anti-RORγt (AFKJS-9); anti–phospho-STAT3 (LUVNKLA); anti-STAT3 (9D8); and anti-IgM (11/41). Anti–GL-7 (GL7); rat anti–mouse CXCR5 (5571961); anti-BCL6 (K112-91); anti-IgG1 (A85-1); anti-IgG2a/2b (R2-40); and anti-IgG3 (R40-82) were purchased from BD Bioscience. SA-APC (S868) was purchased from Life Technologies.

### ELISA.

IL-6 in the supernatant was measured by anti–mouse IL-6 DuoSet (both from R&D Systems), according to the manufacturer’s protocol. IL-21 and IL-17 in the supernatant were measured by Mesoscale Discovery (MSD) U-Plex Th17 Combo Kit (catalog K15078K-1, according to the manufacturer’s protocol.

For Ig ELISA, a 96 well plate (Costar) was coated with 1 μg/mL of goat anti–mouse Ig polyclonal Abs (Southern Biotechnologies) in PBS at 4°C overnight. The next day, the plate was washed with washing buffer (0.05 % Tween 20 in PBS) and blocked with 1% BSA in PBS for 2 hours at 37°C in an incubator. Samples and purified Ig (for standard) were diluted in 0.2% of BSA in PBS and incubated for 1 hour at 37°C. Bound Ig was detected by AP-conjugated anti-Ig (Southern Biotechnologies) in 0.1% BSA in PBS for 1 hour at 37°C. For NP-specific ELISA, 10 μg/mL NP 2-BSA or NP 26-BSA (Bioresearch Technologies), coated plates were prepared. Each plate was developed with p-nitrophenyl phosphate (Sigma), and OD was measured at 405 nm by Victor3 (Perkin Elmer).

### Caspase activity assay.

To measure the activity of caspse-1 in the cells, FAM-FLICA caspase assay kit (BioRad) was used and the labeling protocol was followed according to the manufacturer’s instruction with modification. Briefly, T cells were harvested up to 5 × 10^6^ and incubated with 300 μL of 1x FLICA solution for 30 minutes at 37°C. At the end of labeling, 200 μL of propidium iodide (PI) solution, provided in the kit, was added directly into the FLICA solution (final 500 μL) and incubated for 5 minutes at 37°C. Labeled cells were washed with wash buffer (provided in the kit) twice and analyzed by Fortessa using the FL-1 (FLICA) and FL-2 (PI) channels.

### Statistics.

A 1-way ANOVA test or unpaired 2-tailed Student’s *t* test (Mann-Whitney) was used for statistical analysis with GraphPad Prism 9 software. For the comparison with more than 2 groups, the Bonferroni or Dunnett’s corrections were performed. A *P* value less than 0.05 was considered significantly different. No exclusion of sample was done.

### Study approval.

All the experiments were conducted followed the guidance in the Guide for the Care and Use of Laboratory Animals of the National Institutes of Health. The protocol was approved by the committee on the Ethics of Animal Welfare of the FIMR (2009-048 term IV).

## Author contributions

SJK designed the experiments. VK, KL, HT, SHJ, and SJK performed the experiments and analyzed the data. BD and SJK wrote the manuscript. KL performed bioinformatic analyses. All authors reviewed and approved the manuscript.

## Supplementary Material

Supplemental data

## Version 1 06/08/2022

Electronic publication
