# Supplementary material for: IL-17–producing follicular Th cells enhance plasma cell differentiation in lupus-prone mice
Source: JCI Insight. 2022 Jun 8;7(11):e157332. doi: 10.1172/jci.insight.157332 (PMC9220957; doi:10.1172/jci.insight.157332)
Supplement: Supplemental data [file jciinsight-7-157332-s191.pdf]

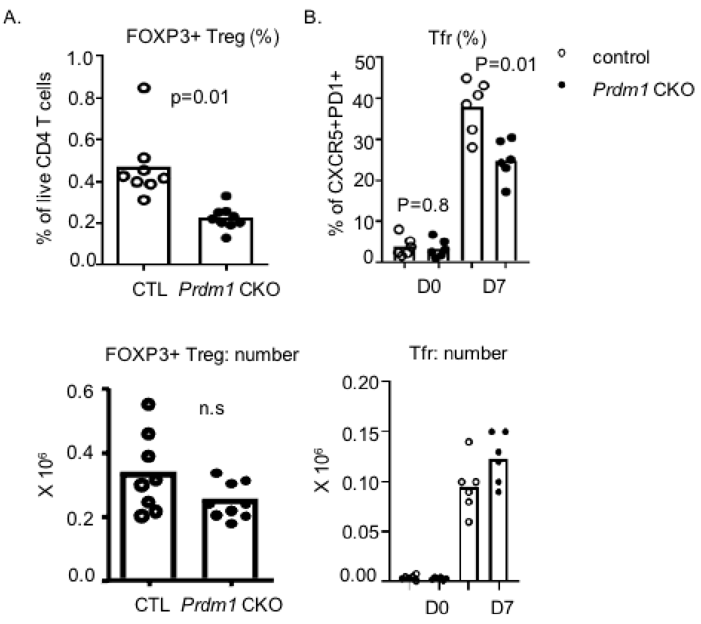

Supplemental figure 1. Frequency and number of Treg and Tfr cells in *Prdm1* CKO mice. **(A)** 6-8 weeks old female FOXP3-GFP *Prdm1* CKO mice and gender/ age-matched littermate control mice were used to investigate FOXP3+ CD4+ Treg cells in the spleens. Percent and number of Treg cells were calculated and plotted. **(B)** To induce Tfr cells, 6-8 weeks old female FOXP3-GFP *Prdm1* CKO mice and gender and age-matched littermate control mice were immunized with NP-CGG (100 µg) in CFA i.p. Unimmunized group (D0) and immunized group (D7) of mice were sacrificed and Tfr cells were identified by flow cytometry analysis. Each dot represents an individual mouse, and the bar graph indicates mean (n=3).

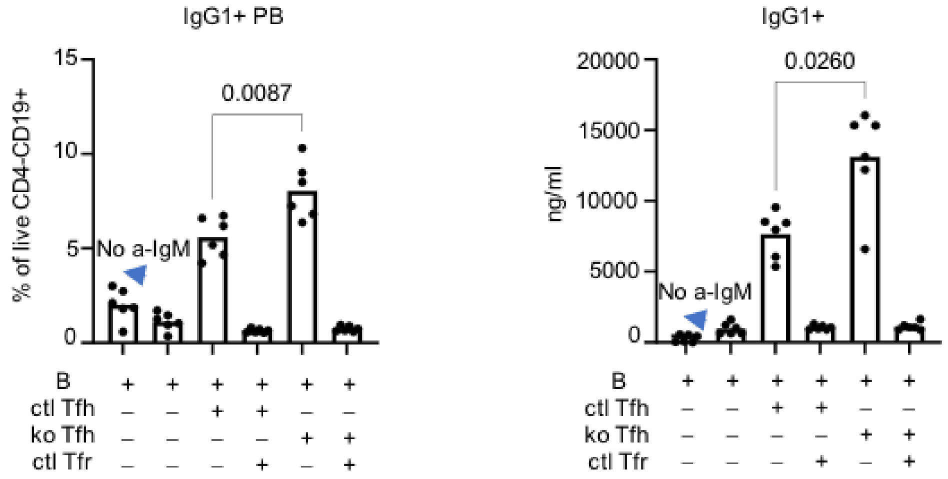

Supplemental figure 2. Increased IgG1+ PB differentiation by Tfh cells from *Prdm1* CKO mice. 6-8 weeks old female FOXP3-GFP; *Prdm1* CKO or FOXP3-GFP; control mice were immunized with NP-CGG s.c. and B cells (CD19+), Tfh (TCRβ+CD4+CXCR5+PD1+FOXP3-) and Tfr (TCRβ+CD4+CXCR5+PD1+FOXP3+) cells were isolated from draining lymph nodes by cell sorter. Isolated B cells were cultured alone, with Tfh or with Tfh and Tfr in the presence of anti-IgM+ anti-CD3 as indicated in the figure except the B alone with blue arrow (no anti-IgM or anti-CD3 was added). After 7 days of culture, PB was identified by staining of GL7, IgG1 and IgM and calculated **(A). (B)** Secreted IgG1 in the supernatant was measured by ELISA. Each dot represents an individual mouse, and the bar represents the mean (n=3).

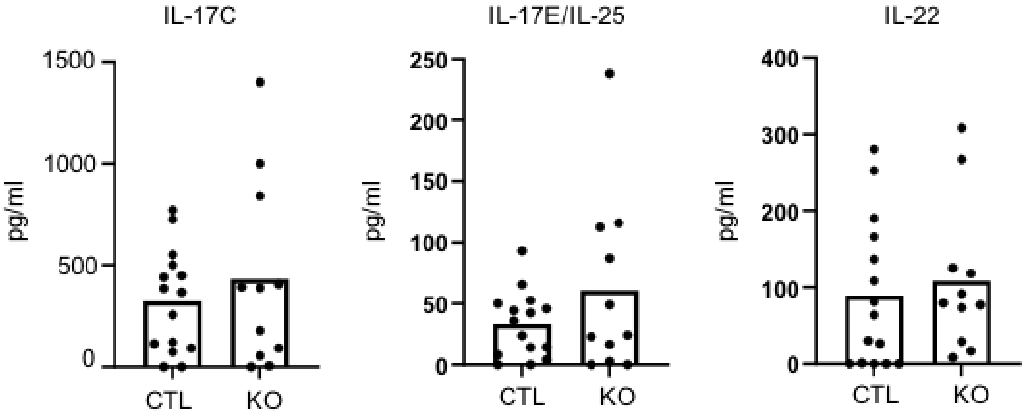

Supplemental figure 3. IL-17 family cytokines secreted from Tfh cells. Spleens were collected from day 7 post NP-immunized *Prdm1* CKO or littermate CTL mice. Tfh cells were isolated and cultured overnight. Next day, the supernatant was collected and measured by MSD. The bar represents the mean, and each dot represents an individual mouse (n=5).

Supplemental table 1. Increased expression in Tfh of Prdm1 CKO mice compared to CTL mice.

Gene	baseMean	log2FC	lfcSE	stat	pvalue	padj
Ighv5-9-1	115.758005	5.05964362	0.7882195	6.41907948	1.371E-10	2.0524E-06
Igkv5-48	31.1802716	6.28135853	1.22329998	5.1347655	2.825E-07	0.00211448
Gm35028	17.2944547	7.30166045	1.5344246	4.75856583	1.9497E-06	0.00972917
Ighv5-9	38.6841584	8.46425119	1.81052278	4.67503159	2.9391E-06	0.01050597
Igkv1-122	15.5135705	7.14806754	1.54521361	4.62594137	3.729E-06	0.01050597
Ighv1-50	19.2635452	7.46457817	1.62248878	4.60069632	4.2108E-06	0.01050597
Ighv7-1	403.119884	1.74536412	0.41308999	4.22514265	2.3879E-05	0.05106683
Ighv1-58	42.8386354	7.61053888	1.85143341	4.11061983	3.946E-05	0.07383923
Hpse	416.852034	0.85180787	0.21636537	3.9368956	8.2542E-05	0.09560757
Ighv2-9-1	13.2748078	6.92661365	1.76003691	3.9354934	8.3026E-05	0.09560757
Fgfbp3	9.30270864	6.40663165	1.75290563	3.65486399	0.00025732	0.20597341
Igkv12-41	43.3441099	6.48747473	1.79914505	3.60586531	0.00031111	0.22178018
Fem1c	621.096862	0.67300462	0.1886287	3.56788023	0.00035988	0.24488261
Iglc1	950.444704	1.63597395	0.46791961	3.49627139	0.00047181	0.30708584
4930452B06Rik	14.8607292	4.1798241	1.28155958	3.26151367	0.00110819	0.47391982
Hace1	268.673208	1.11046392	0.34053897	3.26090114	0.00111059	0.47391982
Bub1b	934.463895	0.74642355	0.22909257	3.25817446	0.00112131	0.47391982
Stbd1	18.0200486	4.25815237	1.31132691	3.24720886	0.00116543	0.47391982
Iglv1	1159.43301	1.57104796	0.48585903	3.23354687	0.00122263	0.47391982
Snx18	215.112881	0.90511847	0.28120874	3.21867123	0.00128786	0.47391982
Col24a1	7.04318563	6.01202903	1.9059871	3.15428632	0.00160891	0.5207021

Cdk16	467.852867	1.04983606	0.33492293	3.13456014	0.00172112	0.52581962
Adam12	6.94341166	5.98327377	1.9485796	3.07058216	0.00213642	0.59226281
Igkv4-80	14.5410609	7.05227447	2.30865564	3.05471044	0.00225278	0.5935101
Neil2	6.69484445	5.93696788	1.95121093	3.04270942	0.00234459	0.5935101
Igkv3-2	49.0763616	4.32226868	1.42156303	3.04050443	0.00236182	0.5935101
Cep131	42.8916214	2.18725563	0.72977364	2.99716995	0.00272499	0.62758558
Il17re	13.1467253	4.77024533	1.59642683	2.9880764	0.00280739	0.62908205
Gm10382	23.4335887	2.13176734	0.72253524	2.95039912	0.00317364	0.68601506
Isg20l2	403.113017	0.70012452	0.23852288	2.93525098	0.00333278	0.69644044
Hbb-bs	144.751611	1.86589952	0.63602533	2.93368744	0.00334961	0.69644044
Slc30a5	548.631923	0.92615417	0.32383924	2.85991953	0.00423749	0.82383315
Rcbtb1	709.85942	0.68006515	0.2399054	2.83472211	0.00458656	0.88026599
Ighv1-19	42.2040267	3.37154751	1.20592944	2.79580827	0.00517701	0.93763336
Ube2l6	441.784416	0.625549	0.22530469	2.77645797	0.00549547	0.93763336
Msl2	372.78232	0.65703013	0.23668744	2.77594006	0.00550424	0.93763336
Igkv9-129	9.55917563	6.44174704	2.32100922	2.77540778	0.00551325	0.93763336
Il23r	7.12371747	5.04952772	1.82647132	2.76463564	0.00569864	0.93763336
Ighv2-7	11.739064	6.74689776	2.44342131	2.76125028	0.00575805	0.93763336
Timm29	545.723275	1.15199913	0.41777328	2.75747444	0.00582498	0.93763336
Ighv14-2	28.7867807	3.99944957	1.46090767	2.73764705	0.00618804	0.93841299
Dnph1	160.202193	0.98242732	0.3594173	2.73338905	0.00626862	0.93841299
Trim62	35.637321	1.59722168	0.58576522	2.72672671	0.0063966	0.94241503
Lmo1	9.95426607	4.35599128	1.60190393	2.71925876	0.00654284	0.95093513
Cd5l	214.766826	0.91614302	0.34101333	2.68653141	0.00721982	0.99622089

Cd109	24.0937774	3.39263399	1.26926208	2.6729184	0.00751945	0.99704377
Sirpa	2001.55681	0.90870081	0.34120267	2.6632289	0.00773947	0.99704377
Gramd1a	2934.17401	0.42663084	0.16027426	2.66187994	0.00777056	0.99704377
Pif1	262.073531	0.9102622	0.34230092	2.65924556	0.00783159	0.99704377
Ighv1-15	47.546926	4.07535745	1.54143602	2.64387065	0.0081964	0.99840724
Sass6	131.183055	1.03913122	0.39487515	2.63154375	0.00849979	0.99840724
Fam105a	2534.60298	0.67042297	0.25492123	2.62992205	0.00854044	0.99840724
Gns	797.181134	0.61008363	0.23228385	2.62645736	0.00862788	0.99840724
Igkv1-88	6.94410409	5.979015	2.28504831	2.61658144	0.00888152	0.99840724
Igkv12-98	9.87187214	6.49160951	2.48395146	2.61342044	0.0089641	0.99840724
1700067K01Rik	5.82088422	5.72623121	2.19471916	2.60909519	0.0090782	0.99840724
C1qc	304.553293	1.4758551	0.56821677	2.5973452	0.00939475	0.99840724
Pls3	28.8167884	2.31166559	0.9053465	2.55334901	0.01066925	0.99840724
Ubqln1	717.552015	0.67981081	0.26758131	2.54057663	0.01106698	0.99840724
Slc26a2	818.670112	0.41576057	0.163794	2.53831387	0.0111388	0.99840724
Uhrf1	1238.97269	0.40244998	0.15942303	2.52441554	0.01158908	0.99840724
Gm4673	21.2128369	2.26723342	0.8998692	2.51951441	0.01175168	0.99840724
Cenpn	178.944402	0.87533074	0.34788732	2.51613292	0.01186504	0.99840724
Igkv6-13	5.47853485	5.65289617	2.2607307	2.50047304	0.01240276	0.99840724
Bex3	477.126397	0.54627267	0.21878395	2.49685898	0.01252988	0.99840724
Zfp622	201.13735	1.15893476	0.46428567	2.49616742	0.01255433	0.99840724
Gm10655	7.51630682	4.46432982	1.79972051	2.4805684	0.01311731	0.99840724
Iglv2	761.261574	0.80156667	0.32420493	2.47240746	0.01342065	0.99840724
Olr1	16.1889427	2.90268294	1.17576646	2.46875807	0.01355829	0.99840724

Abcb10	58.4332462	1.24107849	0.50629378	2.4513011	0.01423408	0.99840724
Spi1	1402.58112	0.97334842	0.39714557	2.45086061	0.01425151	0.99840724
Ccnb1	679.984307	0.59352131	0.24492047	2.42332256	0.01537926	0.99840724
Iglc2	1011.7235	0.91460274	0.37849715	2.4164059	0.01567458	0.99840724
Iglc3	388.460495	1.39841705	0.58228438	2.40160495	0.01632333	0.99840724
Rxrb	259.021076	0.59948775	0.2497494	2.40035715	0.01637908	0.99840724
Slc9a3r2	8.60680537	3.74526048	1.56328531	2.39576259	0.01658583	0.99840724
Zfyve9	137.457905	0.71869258	0.30032598	2.39304167	0.01670934	0.99840724
Ighd	215.618784	0.86194283	0.36101778	2.3875357	0.01696176	0.99840724
Ly6icdkIl23	276.0454	1.03068777	0.43171499	2.38742643	0.0169668	0.99840724
Asf1b	901.158175	0.37745533	0.15876183	2.37749428	0.01743071	0.99840724
Klhl14	4.59859184	5.39855985	2.28388075	2.36376608	0.01809023	0.99840724
Dnajb2	142.752032	0.66044715	0.2795012	2.36294926	0.01813015	0.99840724
Igkv6-14	8.59672358	6.29040461	2.6764625	2.35026817	0.01875989	0.99840724
Csgalnact2	259.757089	1.17063523	0.49822558	2.34960887	0.01879315	0.99840724
Khdc3	8.63749607	4.17857836	1.7998523	2.32162292	0.02025325	0.99840724
Gm16124	27.9312404	1.77197398	0.76378008	2.32000549	0.02034058	0.99840724
Zfp318	73.4419718	1.3103986	0.5650822	2.31895219	0.02039763	0.99840724
Oxsr1	271.861136	0.72551405	0.31326338	2.31598747	0.02055895	0.99840724
Ptafr	298.52026	1.17195143	0.50847039	2.30485678	0.0211746	0.99840724
4930579K19Rik	8.14316684	3.66583902	1.59251736	2.30191463	0.02133999	0.99840724
Gtf2h4	347.643044	0.72599881	0.31546786	2.30133998	0.02137242	0.99840724
Pgp	322.351234	0.69510739	0.30214123	2.30060426	0.02141401	0.99840724
Pacsin2	783.969578	0.53673876	0.23346127	2.29904839	0.02150219	0.99840724

Jchain	4779.01869	0.6000044	0.26185118	2.29139463	0.0219406	0.99840724
BC037032	24.1921289	1.64461338	0.72084762	2.28149937	0.02251891	0.99840724
Ccdc117	396.988297	0.6508291	0.28545882	2.27994043	0.02261122	0.99840724
Acsl3	62.6690585	1.174448	0.51604554	2.27586115	0.02285432	0.99840724
Ighv3-8	11.9645062	3.46879706	1.52975296	2.26755376	0.02335642	0.99840724
Rhbdf1	9.6699625	3.00997089	1.32842797	2.26581415	0.02346276	0.99840724
Tmco6	308.724628	0.67998465	0.30189107	2.25241727	0.02429592	0.99840724
Asic1	8.10926985	3.66689109	1.63021907	2.24932412	0.02449188	0.99840724
Gm4798	6.13393905	4.17136515	1.85601571	2.24748375	0.02460913	0.99840724
Mark4	90.9806007	1.09842703	0.48885534	2.24693676	0.02464407	0.99840724
Rab30	18.4780576	2.41054811	1.07288869	2.24678303	0.0246539	0.99840724
Uqcr11	513.158441	0.56540996	0.2527246	2.23725731	0.02526953	0.99840724
Gm11674	4.88767781	5.47078701	2.44547302	2.2371079	0.02527929	0.99840724
Igkv3-7	21.5402372	3.30814892	1.4821369	2.23201306	0.0256141	0.99840724
Igkv4-57	32.0169405	2.46433127	1.10576289	2.22862542	0.02583884	0.99840724
Xxylt1	99.1291112	1.12058066	0.50306548	2.22750456	0.02591357	0.99840724
Cenpi	203.053088	0.61018133	0.27435039	2.22409498	0.02614205	0.99840724
Tob1	198.333556	0.68448056	0.30789968	2.22306353	0.02621152	0.99840724
Ighv1-53	54.8159648	1.44731648	0.65507929	2.20937602	0.0271485	0.99840724
Hectd3	770.61753	0.50806454	0.23145754	2.19506579	0.02815888	0.99840724
Tusc2	346.782066	0.84759279	0.38703617	2.18995757	0.02852731	0.99840724
Sod2	1043.06487	0.41535983	0.18984193	2.18792461	0.02867509	0.99840724
Il13ra1	510.735346	0.70061117	0.320571	2.18551015	0.02885146	0.99840724
Sqle	400.303623	0.63722476	0.29160064	2.18526534	0.0288694	0.99840724

Atp13a2	794.668218	0.57706128	0.26450314	2.1816803	0.02913314	0.99840724
Ngp	239.560588	1.09405741	0.50191109	2.17978329	0.02927353	0.99840724
Iglj1	80.8974019	1.25002606	0.57500194	2.17395104	0.02970881	0.99840724
Siglecg	185.612166	0.78868726	0.36344733	2.17001805	0.03000548	0.99840724
Gch1	266.691286	0.98429858	0.45510234	2.1628071	0.03055602	0.99840724
Ankrd29	25.3839643	1.76578688	0.82062154	2.15176765	0.03141566	0.99840724
Cyp2d22	29.0534434	1.58001033	0.73537457	2.14857898	0.03166779	0.99840724
Gm31024	28.7899092	1.33704748	0.62294721	2.14632552	0.03184702	0.99840724
5830416I19Rik	47.6477454	1.2495466	0.58255067	2.1449578	0.03195622	0.99840724
Tmem177	172.431342	0.72286647	0.33706306	2.14460305	0.0319846	0.99840724
Arhgap39	189.9317	0.64607865	0.30142622	2.14340558	0.03208055	0.99840724
Wscd1	4.43177877	5.33916135	2.49936349	2.13620843	0.03266243	0.99840724
Thap11	457.563662	0.82780343	0.38762722	2.13556578	0.03271482	0.99840724
Prim2	432.174206	0.49078297	0.22997907	2.13403319	0.03284006	0.99840724
Igkv14-130	11.3694214	6.69598472	3.13788488	2.13391663	0.03284961	0.99840724
Ighv1-31	11.3694214	6.69598472	3.13788488	2.13391663	0.03284961	0.99840724
Gas2l3	113.007969	0.96859157	0.45465058	2.13040873	0.03313788	0.99840724
Rps4l	32.9783915	2.48372444	1.16597018	2.13017835	0.03315689	0.99840724
Wfs1	90.1513817	0.91454242	0.42962948	2.12867709	0.03328099	0.99840724
Pcdhga5	5.84996507	4.10338542	1.93056225	2.12548724	0.03354598	0.99840724
Ighg2b	52.9495229	2.75255469	1.29603923	2.1238205	0.03368516	0.99840724
Ndfip2	413.388578	0.55980345	0.26408637	2.11977412	0.0340251	0.99840724
Hba-a1	48.9209582	1.28962948	0.6087473	2.11849725	0.03413298	0.99840724
5031434O11Rik	30.0714409	1.83980056	0.87137343	2.11138015	0.03473965	0.99840724

Ighv9-1	7.33653089	6.06838679	2.87567412	2.11024843	0.03483696	0.99840724
Iglj3	40.9515212	1.73605808	0.82370106	2.10763123	0.0350629	0.99840724
Mcur1	521.944156	0.78279843	0.37290331	2.09919947	0.03579932	0.99840724
Ddx3x	1946.20215	0.37718942	0.1798496	2.09724909	0.03597153	0.99840724
B3galt6	208.065103	0.65331219	0.31160924	2.09657515	0.0360312	0.99840724
Slc30a9	480.383583	0.66003166	0.31481476	2.09657153	0.03603152	0.99840724
Derl3	80.7332274	1.11412201	0.53147706	2.09627487	0.03605781	0.99840724
Inppl1	107.760983	1.01182072	0.48339086	2.09317303	0.03633371	0.99840724
Ccrl2	182.723012	0.57835248	0.2770039	2.08788567	0.03680815	0.99840724
Ttc12	7.50241976	3.55565884	1.70548229	2.08484067	0.03708377	0.99840724
Mycbp	121.869869	0.87951484	0.42189499	2.08467712	0.03709862	0.99840724
Cd300lf	172.444852	0.89094967	0.42750721	2.08405764	0.03715492	0.99840724
Dscc1	112.266881	0.7232688	0.34747575	2.08149436	0.03738868	0.99840724
Bex6	28.499711	1.83412794	0.88194896	2.07963048	0.03755944	0.99840724
Nphp1	17.0900367	2.50986668	1.21446351	2.06664643	0.03876748	0.99840724
Epha4	20.6175206	2.44453547	1.18304237	2.06631269	0.03879896	0.99840724
Wdr36	857.697595	0.55730536	0.26982967	2.0653969	0.03888546	0.99840724
Fbrsl1	196.892591	0.85658333	0.41586001	2.05978766	0.03941884	0.99840724
Ighd1-1	29.2147127	1.51149631	0.7344826	2.05790622	0.03959914	0.99840724
E330021D16Rik	5.23004074	3.94781123	1.9184458	2.05781745	0.03960766	0.99840724
Gm30238	3.68445147	5.07628378	2.46966835	2.0554516	0.03983541	0.99840724
Phldb1	33.5457992	1.26625573	0.61994306	2.04253555	0.04109844	0.99840724
Gm43339	20.311789	1.89426092	0.93045869	2.0358356	0.04176687	0.99840724
Prr7	5.8455944	5.73338592	2.82829208	2.02715482	0.04264658	0.99840724

Slc37a2	172.123502	0.7142661	0.35359563	2.02000829	0.04338253	0.99840724
Car9	21.9914294	2.48890279	1.23677301	2.0124168	0.04417602	0.99840724
Lancl3	84.5390048	0.89695868	0.44735033	2.00504751	0.04495797	0.99840724
St8sia6	359.393618	0.68368694	0.34118152	2.00388037	0.04508288	0.99840724
Soat1	1285.95687	0.50461065	0.25194318	2.00287484	0.04519072	0.99840724
Igkv4-61	24.7282299	7.81919125	3.90827243	2.00067713	0.0454272	0.99840724
Slc27a4	76.5821205	1.55057692	0.77829546	1.99227286	0.04634113	0.99840724
Zfp934	50.9128696	1.44419271	0.72523034	1.99135726	0.04644162	0.99840724
Plcd1	13.8194254	2.89718475	1.45542062	1.9906168	0.04652303	0.99840724
Rps18-ps3	58.7449418	0.88761114	0.4465017	1.98792332	0.04682017	0.99840724
Sdc3	365.921729	0.7267453	0.36629389	1.98404976	0.04725029	0.99840724
Tspan9	71.7787747	0.97272757	0.49070942	1.98228838	0.04744697	0.99840724
Ppp4c	2909.36726	0.58160806	0.29394686	1.97861632	0.04785922	0.99840724
Tnfrsf13c	66.8939873	1.08829091	0.55023255	1.97787446	0.04794287	0.99840724
2610306M01Rik	33.5468813	1.32894502	0.673086	1.97440598	0.0483356	0.99840724
Sumf1	391.121578	0.90279527	0.45875411	1.96792847	0.04907627	0.99840724
Gm37593	18.776116	2.42847455	1.23603982	1.96472194	0.04944643	0.99840724
Gm37893	15.8542124	1.80217252	0.91749988	1.96422098	0.04950447	0.99840724
Hacd2	191.811166	0.69890743	0.35598778	1.96329051	0.04961243	0.99840724
F11r	37.3696237	1.17969282	0.60089372	1.96323042	0.04961941	0.99840724

Supplemental table 2. Decreased expression in Tfh of Prdm1 CKO mice compared to CTL mice.

Gene	baseMean	log2FC	lfcSE	stat	pvalue	padj
Igkv4-70	16.9614287	-7.8190248	1.91522804	-4.0825555	4.4543E-05	0.07409013
Prps1l1	21.6000602	-3.7016003	0.92852404	-3.9865422	6.7043E-05	0.09560757
Nbas	308.369534	-0.8372288	0.21147732	-3.958953	7.5279E-05	0.09560757
Tmem8b	44.6664942	-2.695158	0.72022835	-3.7420882	0.0001825	0.19188449
Kmt2d	4888.60202	-0.6948023	0.18632584	-3.7289635	0.00019227	0.19188449
Gm39321	30.2113925	-5.8232136	1.57203733	-3.7042464	0.00021202	0.19837134
Sorbs1	215.275546	-0.9496841	0.25899745	-3.66677	0.00024563	0.20597341
Ighv1-36	37.9794054	-5.6344549	1.54871153	-3.6381565	0.0002746	0.20597341
Slc5a3	374.837993	-0.8829601	0.24273095	-3.6376082	0.00027518	0.20597341
Gm45251	20.0176274	-4.9630393	1.42723778	-3.4773738	0.00050635	0.31499559
Ddc	112.266318	-1.2305464	0.3550112	-3.4662186	0.00052783	0.31499559
Ccl5	784.048874	-0.8261983	0.23927264	-3.4529577	0.00055448	0.31499559
Rgs11	179.795405	-0.939038	0.27246998	-3.4463907	0.00056813	0.31499559
Cyp1a1	38.7732147	-2.6466584	0.78351622	-3.3779242	0.00073035	0.39047756
Cldn10	90.4423241	-1.2280507	0.36723833	-3.3440156	0.00082575	0.4262583
Ahrr	31.7030662	-4.4937373	1.35525985	-3.3157754	0.00091389	0.4527609
Rbm44	41.3346887	-1.7833891	0.53901352	-3.3086167	0.00093758	0.4527609
Fcer1a	76.1921106	-1.5742713	0.48262046	-3.261924	0.00110659	0.47391982
Gm18290	9.24790672	-6.9378229	2.15092324	-3.2255093	0.00125749	0.47391982
Gm42636	38.4841614	-3.198217	0.9930877	-3.2204779	0.00127977	0.47391982
Gzma	344.300529	-1.1542184	0.35945518	-3.2110217	0.00132264	0.47391982

Mysm1	1388.12833	-0.514869	0.16055411	-3.2068252	0.00134209	0.47391982
Ulk2	260.965349	-0.8499305	0.26585908	-3.1969212	0.00138903	0.47391982
Cd200r3	159.347172	-1.2480797	0.39049989	-3.1961078	0.00139295	0.47391982
Gm26732	213.02059	-0.8719369	0.27637847	-3.1548654	0.00160572	0.5207021
A530041M06Rik	176.976118	-1.4761406	0.4686719	-3.1496248	0.0016348	0.5207021
Zfp407	281.756477	-0.8667542	0.27616711	-3.1385133	0.00169807	0.52581962
Gm16107	10.1779264	-6.0967184	1.95657425	-3.1160169	0.00183312	0.54883548
Gm37655	29.6511863	-4.6382711	1.49282422	-3.1070444	0.00188968	0.55467677
Gm38125	11.325855	-4.5435037	1.47373626	-3.0829829	0.00204937	0.58276461
Ap1s3	33.2507639	-1.7717624	0.57506517	-3.0809767	0.00206323	0.58276461
Rnf24	69.7114449	-2.4523854	0.80169252	-3.05901	0.0022207	0.5935101
Gm29112	31.0923762	-3.77255	1.23799432	-3.047308	0.00230901	0.5935101
Edaradd	42.2314696	-1.8669919	0.61482521	-3.0366222	0.00239245	0.5935101
Vps13c	405.084437	-0.9948876	0.32806797	-3.0325655	0.00242484	0.5935101
Siglec5	27.0431463	-2.4132368	0.80407967	-3.0012409	0.00268882	0.62758558
Mfsd4a	262.571534	-1.3523637	0.45103481	-2.9983578	0.00271439	0.62758558
Selp	25.6194564	-3.7330591	1.24968849	-2.9871917	0.00281553	0.62908205
Nlrc5	2529.827	-0.4986532	0.1672505	-2.9814751	0.00286863	0.63152129
Gm26947	12.9861251	-6.4573535	2.19109629	-2.947088	0.00320782	0.68601506
Utrn	5638.09225	-0.6217013	0.21339186	-2.9134258	0.00357487	0.7330928
Mypopos	336.309577	-0.8054994	0.27785585	-2.8989828	0.00374375	0.75735129
Zc2hc1c	23.3579764	-2.7855122	0.96592671	-2.8837718	0.00392943	0.78431496
Ggt1	74.4093776	-1.6358382	0.56993867	-2.8702005	0.00410212	0.80800902
Gm43677	13.4616607	-4.8366909	1.71309533	-2.8233635	0.00475227	0.90052419

AC122035.2	15.6369818	-3.08665	1.10417161	-2.7954441	0.00518285	0.93763336
Gm9938	17.5241823	-3.4428343	1.232487	-2.7934042	0.00521565	0.93763336
Tmod1	45.3311499	-2.2127888	0.79375795	-2.7877375	0.00530775	0.93763336
Patl2	22.1270942	-2.5178548	0.90467407	-2.7831623	0.00538319	0.93763336
Stx3	110.968299	-1.2026524	0.43407204	-2.7706285	0.00559482	0.93763336
1700019J19Rik	8.84220852	-5.1534757	1.86635517	-2.7612513	0.00575803	0.93763336
Ogt	4411.57091	-0.504779	0.18296391	-2.7588992	0.00579964	0.93763336
Dip2c	23.3604032	-2.5676871	0.93599848	-2.7432599	0.00608325	0.93841299
Gm10801	77.8882276	-2.7189016	0.99172166	-2.7415974	0.00611412	0.93841299
Sdccag8	513.651651	-0.6961144	0.25396092	-2.7410294	0.0061247	0.93841299
Gm7967	35.7804051	-3.1662995	1.15696862	-2.7367203	0.0062055	0.93841299
Gramd1c	94.4573564	-1.7435261	0.63714154	-2.7364815	0.00621001	0.93841299
Slc20a1	1333.81995	-0.553335	0.20302472	-2.7254564	0.00642126	0.94241503
Igkv11-125	8.27496562	-6.7901267	2.50253953	-2.7132945	0.00666179	0.95783036
Gm36955	32.4969766	-2.8430531	1.04922786	-2.7096622	0.00673518	0.95783036
Ighv1-4	8.46907317	-4.0087395	1.48068654	-2.7073519	0.00678223	0.95783036
Psd3	27.9038188	-2.0678032	0.76607039	-2.699234	0.00694993	0.97234065
Gm37531	128.838468	-1.3667123	0.50938332	-2.6830723	0.00729492	0.99622089
Wdr60	34.4265097	-2.0929179	0.7803826	-2.6819125	0.00732026	0.99622089
Ipcef1	1492.75196	-0.7944045	0.29718498	-2.6730976	0.00751544	0.99704377
Kcnab1	24.4718483	-2.5037515	0.93870094	-2.6672515	0.00764744	0.99704377
Mtor	407.386616	-0.4987246	0.18758872	-2.6586067	0.00784645	0.99704377
Gm44661	14.2559125	-3.5858989	1.34912593	-2.6579423	0.00786193	0.99704377
Ahdc1	128.610736	-0.8846955	0.33319137	-2.6552174	0.00792573	0.99704377

Ighv5-17	238.888362	-1.5713886	0.59579735	-2.6374549	0.00835307	0.99840724
Abcb7	318.047276	-0.7084508	0.26922803	-2.6314154	0.008503	0.99840724
Fxyd4	34.1043789	-2.1013551	0.80112	-2.6230217	0.00871537	0.99840724
Gm45407	15.9276145	-2.9780482	1.13606716	-2.6213663	0.00875781	0.99840724
Rsl1	231.942078	-0.7193917	0.27531374	-2.6129886	0.00897543	0.99840724
Strc	6.28681625	-4.647832	1.78529631	-2.6033953	0.00923055	0.99840724
Kif1b	679.801641	-0.6731311	0.25966618	-2.5922941	0.00953382	0.99840724
Ighv2-2	104.901205	-1.4330906	0.55569927	-2.5788958	0.00991167	0.99840724
Slc2a4rg-ps	684.169281	-0.5353207	0.20935301	-2.5570239	0.0105572	0.99840724
Gm48138	25.7479376	-2.322956	0.91166716	-2.5480308	0.01083329	0.99840724
Cd28	4684.44757	-0.370443	0.14555128	-2.545103	0.01092455	0.99840724
Epn2	34.6532549	-2.3407623	0.9243763	-2.5322612	0.01133295	0.99840724
A930024E05Rik	84.0906746	-0.9375064	0.37036269	-2.5313199	0.01136342	0.99840724
Tmem267	19.6125133	-2.3856181	0.94378916	-2.5277024	0.01148116	0.99840724
Secisbp2	774.452536	-0.5372006	0.21280508	-2.5243785	0.0115903	0.99840724
9430018G01Rik	10.9378933	-5.4895039	2.17979397	-2.5183591	0.01179031	0.99840724
Pibf1	314.306454	-0.8999261	0.35736026	-2.5182601	0.01179362	0.99840724
Ms4a2	172.421249	-1.5998607	0.63667691	-2.5128298	0.01197671	0.99840724
Dnajc16	201.795012	-0.6927238	0.27584544	-2.5112752	0.01202959	0.99840724
Josd1	193.747549	-1.3537592	0.53991352	-2.5073631	0.01216357	0.99840724
Gm43421	17.4107253	-2.8441393	1.13682246	-2.5018324	0.01235524	0.99840724
Ramp3	93.306793	-1.1608502	0.46462809	-2.4984502	0.01247377	0.99840724
Ranbp2	874.411308	-0.7599332	0.30523224	-2.4896884	0.01278551	0.99840724
Gm26551	263.672586	-1.0948356	0.440152	-2.4874034	0.01286794	0.99840724

Ccl4	228.835877	-1.0816041	0.43595721	-2.4809869	0.01310192	0.99840724
Agl	140.972867	-1.0930985	0.44081223	-2.4797372	0.01314793	0.99840724
Plod1	250.51639	-0.7527149	0.30405288	-2.4756051	0.01330106	0.99840724
Zfp738	268.701592	-0.5987956	0.24285586	-2.4656419	0.0136768	0.99840724
Inpp4b	4210.99399	-0.5796571	0.23565681	-2.4597509	0.01390335	0.99840724
Gm48855	65.8593189	-1.4134949	0.57507183	-2.4579449	0.01397346	0.99840724
Gm40309	285.563814	-0.8340056	0.33952013	-2.4564246	0.01403272	0.99840724
Gon4l	195.343404	-0.8427945	0.34317552	-2.4558701	0.01405439	0.99840724
9530085L11Rik	21.2218235	-2.6526404	1.08221396	-2.4511239	0.01424109	0.99840724
Mical2	74.6749897	-1.4561454	0.59419521	-2.4506179	0.01426113	0.99840724
Gm43336	78.0822455	-1.4482645	0.59119014	-2.449744	0.01429578	0.99840724
Gm37906	98.5802799	-1.6270941	0.66439133	-2.4489997	0.01432536	0.99840724
Adamts14	53.7611846	-2.2269479	0.91089054	-2.444803	0.01449313	0.99840724
Dock8	1608.20557	-0.4408688	0.18060947	-2.441006	0.01464641	0.99840724
Lypd6b	164.131411	-0.9708797	0.39809938	-2.4387872	0.01473664	0.99840724
Slmap	337.56977	-0.8575688	0.35251671	-2.432704	0.01498655	0.99840724
Sytl2	396.826593	-0.8264915	0.33980075	-2.4322827	0.01500399	0.99840724
Gm10388	18.3605513	-2.6658437	1.0968197	-2.4305214	0.01507712	0.99840724
Jag2	58.5207753	-2.1820143	0.90164452	-2.4200383	0.01551887	0.99840724
Ankrd44	2542.55626	-0.4863623	0.20116765	-2.4176965	0.0156191	0.99840724
Gm43589	7.38856567	-6.6189784	2.74574226	-2.4106336	0.01592484	0.99840724
Zfp949	322.481174	-0.563633	0.23405981	-2.4080725	0.016037	0.99840724
Gm48314	21.5772469	-2.1253709	0.88264175	-2.4079655	0.0160417	0.99840724
Ice1	505.371415	-0.9673703	0.40188667	-2.4070723	0.01608099	0.99840724

Trbv29	107.642108	-0.9008787	0.37452407	-2.4053959	0.01615495	0.99840724
Pcdhgc4	52.8562182	-1.9358218	0.80553824	-2.4031407	0.01625493	0.99840724
Gm47585	200.543166	-1.1046758	0.45970705	-2.4029995	0.01626121	0.99840724
Nmt2	926.307618	-0.611684	0.25487181	-2.3999674	0.01639653	0.99840724
Gm45884	38.6526984	-2.1355035	0.89069172	-2.3975787	0.01650383	0.99840724
D130062J10Rik	42.1992788	-2.0229221	0.84479935	-2.3945593	0.01664035	0.99840724
Sla2	896.496774	-0.4050759	0.16923514	-2.3935685	0.01668537	0.99840724
Tmem185b	648.914537	-0.5875923	0.2462023	-2.3866238	0.01700388	0.99840724
Gm48624	41.2013858	-2.2112087	0.92770101	-2.383536	0.01714721	0.99840724
Gm5122	11.4664655	-5.5714108	2.3400491	-2.3808948	0.01727064	0.99840724
6030400A10Rik	16.7260472	-2.3662575	0.99494452	-2.3782809	0.01739357	0.99840724
Gm18646	34.5873569	-2.2020397	0.93555453	-2.3537268	0.01858627	0.99840724
Gm42989	6.94255958	-6.5291258	2.77557044	-2.3523546	0.01865498	0.99840724
AC154562.1	5.36958915	-5.1607673	2.20098064	-2.3447582	0.01903942	0.99840724
Smyd4	349.620507	-0.6614441	0.28221916	-2.3437249	0.01909225	0.99840724
Ranbp10	439.11948	-0.6862196	0.29293286	-2.3425831	0.01915077	0.99840724
Kif21b	2162.84224	-0.3130098	0.1336638	-2.3417692	0.01919258	0.99840724
Spg11	333.334773	-0.7414733	0.317191	-2.3376241	0.01940676	0.99840724
Tub	24.4090193	-3.042626	1.30305464	-2.3349949	0.01954368	0.99840724
Dennd1c	2765.59536	-0.3594043	0.1539302	-2.3348527	0.01955112	0.99840724
Dnd1	38.8503001	-2.3846603	1.02594614	-2.3243523	0.02010662	0.99840724
Gm13710	16.553112	3.31175025	1.42589276	2.32258017	0.02020172	0.99840724
E230034D01Rik	31.7144779	-1.4694101	0.63292219	-2.3216283	0.02025296	0.99840724
Slc7a8	139.189507	-1.1640979	0.50218958	-2.3180446	0.0204469	0.99840724

Mtg1	359.972546	-0.5292611	0.22877284	-2.3134789	0.02069632	0.99840724
Gm17195	18.6517231	-2.0570916	0.89052845	-2.3099673	0.02088996	0.99840724
Rasl10a	7.23314026	-5.6028484	2.43682082	-2.2992451	0.02149102	0.99840724
Gm37423	124.351854	-1.1411537	0.49718652	-2.2952225	0.02172038	0.99840724
Gm10614	12.2687317	-3.6211576	1.57778113	-2.295095	0.02172768	0.99840724
Gm38118	169.75658	-1.3089128	0.57291636	-2.2846491	0.02233341	0.99840724
Gm16845	37.9174981	-1.4260662	0.62626543	-2.2770954	0.02278053	0.99840724
Gm37598	15.943482	-2.6966982	1.1852859	-2.2751458	0.02289718	0.99840724
Pkd1	701.443876	-0.5470703	0.24050692	-2.274655	0.02292663	0.99840724
Gm44851	10.3839324	-3.8670126	1.70101048	-2.273362	0.02300437	0.99840724
Zfx	420.65795	-0.5294226	0.23292176	-2.2729633	0.02302839	0.99840724
Gm43672	162.172168	-1.0791538	0.47546981	-2.2696579	0.02322835	0.99840724
Hgf	118.453407	-1.0082021	0.44452318	-2.268053	0.02332598	0.99840724
4930444A19Rik	45.3604243	-1.5636092	0.68964963	-2.2672516	0.02337486	0.99840724
Ccdc97	805.336588	-0.3934977	0.17358291	-2.2669149	0.02339543	0.99840724
Rab11fip3	97.6945342	-1.015992	0.44988966	-2.2583137	0.02392611	0.99840724
Gucy1a1	234.496577	-0.6321113	0.27996055	-2.2578583	0.02395449	0.99840724
Gm38387	17.5947728	-2.099311	0.93035805	-2.2564549	0.02404216	0.99840724
Cd8a	19.2562541	-2.050607	0.90908285	-2.2556877	0.02409019	0.99840724
Nr3c1	1097.3664	-0.5678488	0.25196152	-2.2537123	0.02421427	0.99840724
Tpbg	10.0351371	-7.0611036	3.13350956	-2.2534169	0.02423287	0.99840724
Gm37166	13.2350158	-2.9243744	1.30296981	-2.2443915	0.02480722	0.99840724
A730011C13Rik	79.5234695	-1.0157865	0.45376531	-2.2385724	0.02518375	0.99840724
mt-Co1	62897.317	-0.6392328	0.28557155	-2.238433	0.02519283	0.99840724

Tbc1d2b	623.220577	-0.4947272	0.22103089	-2.2382717	0.02520335	0.99840724
Heatr5a	362.479919	-0.9922809	0.44376177	-2.2360666	0.02534741	0.99840724
Kdm3b	628.296946	-0.5823616	0.26060505	-2.2346521	0.0254402	0.99840724
Hsf1	1025.84488	-0.3739532	0.16752962	-2.2321619	0.02560427	0.99840724
Acvr1c	7.96728154	-5.0328307	2.25840065	-2.2284933	0.02584764	0.99840724
2810403D21Rik	59.6123257	-1.2111725	0.54361871	-2.2279815	0.02588175	0.99840724
Gm4759	2028.59807	-0.6387364	0.28677556	-2.2273043	0.02592694	0.99840724
Itga4	4824.3197	-0.4793327	0.21543591	-2.2249433	0.02608505	0.99840724
Zbtb24	672.850602	-0.447973	0.20207517	-2.2168634	0.02663243	0.99840724
Ndrg3	1691.60478	-0.4356026	0.1967554	-2.2139294	0.02683364	0.99840724
Edem3	156.0268	-1.1449727	0.5173461	-2.2131657	0.02688622	0.99840724
Kat6a	1118.07643	-0.5238516	0.23674518	-2.2127235	0.02691671	0.99840724
Gm1966	1991.96617	-0.3689311	0.1667716	-2.2121939	0.02695327	0.99840724
Zbtb3	40.6059493	-1.6003194	0.72350966	-2.211884	0.02697468	0.99840724
Nisch	3058.00664	-0.465888	0.21092498	-2.2087855	0.02718957	0.99840724
Spin4	18.8168323	-2.5400921	1.15026521	-2.2082665	0.0272257	0.99840724
Tmem39b	116.683668	-0.7575137	0.34338118	-2.2060433	0.02738097	0.99840724
Rhoh	4741.23711	-0.2860905	0.1299286	-2.2019058	0.02767197	0.99840724
Macf1	4246.36174	-0.6079467	0.27613585	-2.2016218	0.02769203	0.99840724
Adamts17	7.74427849	-4.9913503	2.26731693	-2.2014348	0.02770526	0.99840724
Trdmt1	235.999482	-0.9514486	0.43228822	-2.2009588	0.02773894	0.99840724
Adamts6	381.61669	-0.9394089	0.42725072	-2.1987298	0.02789714	0.99840724
Zfp831	538.318372	-0.8725576	0.39699363	-2.1979133	0.02795528	0.99840724
Calcrl	235.306806	-0.9717393	0.44255402	-2.195753	0.02810963	0.99840724

Tex15	90.8285743	-1.9524001	0.88952383	-2.1948822	0.02817205	0.99840724
Gm46367	15.825102	-2.4883611	1.13486041	-2.1926583	0.02833201	0.99840724
Mylk2	11.889765	-2.6453501	1.20910953	-2.1878499	0.02868054	0.99840724
Mid2	15.5641635	-3.2140314	1.47015618	-2.1861837	0.02880217	0.99840724
Neb	222.479105	-1.2324345	0.56378386	-2.1860052	0.02881522	0.99840724
Gdpd3	23.2694239	-1.7143538	0.78485697	-2.1842881	0.02894108	0.99840724
Gm45358	7.80812353	-4.0322299	1.84954216	-2.1801232	0.02924833	0.99840724
Gm45191	140.018557	-1.2347258	0.56756447	-2.1754812	0.02959408	0.99840724
Msh3	492.619535	-0.4638092	0.21339463	-2.1734812	0.02974412	0.99840724
Gprin3	874.062188	-0.6395844	0.29432597	-2.173048	0.02977671	0.99840724
Chst2	379.921135	-0.6453494	0.29753809	-2.1689641	0.03008541	0.99840724
Chd7	791.168899	-0.6006034	0.27697102	-2.1684702	0.03012293	0.99840724
Gm13357	13.4457366	-2.3239293	1.0727706	-2.1662872	0.03028924	0.99840724
Cep192	709.782663	-0.4421659	0.20431825	-2.1641038	0.03045638	0.99840724
Ctns	326.458623	-0.526676	0.24344034	-2.1634707	0.030505	0.99840724
Polr2a	1553.50152	-0.4637125	0.21469514	-2.159865	0.03078312	0.99840724
4732491K20Rik	16.7766711	-2.8581495	1.32414966	-2.1584792	0.03089059	0.99840724
Ift172	141.037791	-0.7953097	0.36848686	-2.1583123	0.03090356	0.99840724
Krba1	664.911017	-0.7822439	0.36252143	-2.1577867	0.03094442	0.99840724
Strbp	255.632087	-0.6245917	0.28959388	-2.1567847	0.03102244	0.99840724
2700046G09Rik	6.89316205	-3.3892446	1.57179966	-2.1562828	0.03106159	0.99840724
Ghrl	15.914267	-4.5197677	2.09838453	-2.1539273	0.03124587	0.99840724
Kcnc2	31.3501635	-2.5366811	1.17939241	-2.1508372	0.03148905	0.99840724
Gm45597	10.3420909	-3.2081232	1.49220114	-2.1499268	0.031561	0.99840724

Gm37019	66.7046315	-1.432464	0.66680926	-2.1482365	0.03169497	0.99840724
Suco	537.36573	-0.5785691	0.26942709	-2.1474051	0.03176104	0.99840724
Rabgap1	561.806728	-0.6272519	0.29239368	-2.1452306	0.03193442	0.99840724
Snx29	191.878555	-0.7639418	0.35636703	-2.1436938	0.03205743	0.99840724
Uros	514.075406	-0.5015981	0.23439222	-2.1399947	0.0323552	0.99840724
Fam220a	129.285856	-1.2673634	0.59330701	-2.1361005	0.03267122	0.99840724
mt-Cytb	33162.6723	-0.6034689	0.2826556	-2.1349971	0.03276125	0.99840724
Kiz	395.946704	-0.8715631	0.40863942	-2.1328416	0.03293773	0.99840724
Pipox	24.9695933	-1.5492708	0.72676104	-2.1317472	0.03302763	0.99840724
Gm37510	71.0694511	-1.2444871	0.58401203	-2.1309271	0.03309515	0.99840724
Adk	1447.43721	-0.285097	0.13386532	-2.1297305	0.03319387	0.99840724
Rasgef1a	81.4104833	-0.8831768	0.41482649	-2.1290271	0.03325202	0.99840724
Gm26740	1080.8178	-0.484508	0.22793732	-2.1256196	0.03353495	0.99840724
Fmr1nb	5.45999925	-6.1809629	2.90914153	-2.124669	0.03361424	0.99840724
Gm38244	756.446391	-0.7264342	0.34196145	-2.1243161	0.03364372	0.99840724
Gemin5	539.152248	-0.6753111	0.31825067	-2.1219471	0.03384218	0.99840724
Cd4	10121.698	-0.2960374	0.13962731	-2.1201973	0.03398941	0.99840724
Gm37931	15.9600723	-3.3097486	1.5626712	-2.118007	0.03417447	0.99840724
Nav2	383.88428	-0.7326012	0.34595315	-2.1176313	0.0342063	0.99840724
Slc22a23	23.0090428	-2.9882429	1.41237519	-2.1157571	0.03436547	0.99840724
Zfp282	270.775343	-0.4716006	0.22315346	-2.1133467	0.0345711	0.99840724
Gm17130	28.1245297	-2.0178191	0.95487428	-2.1131777	0.03458556	0.99840724
Esco1	1178.67548	-0.5057314	0.23934218	-2.1130057	0.03460028	0.99840724
Iqsec1	302.442504	-0.6978878	0.330491	-2.1116696	0.0347148	0.99840724

Gm11926	5.70131025	-6.2446807	2.95826845	-2.1109243	0.03477882	0.99840724
Tmem186	461.781234	-0.6631592	0.31417901	-2.1107685	0.03479221	0.99840724
Mturn	200.563783	-1.1071536	0.52562832	-2.1063432	0.03517455	0.99840724
Gm13213	16.7977888	-2.2021409	1.04549796	-2.1063081	0.03517759	0.99840724
Pou2af1	518.324618	-0.599401	0.28459649	-2.1061434	0.0351919	0.99840724
Gm47358	8.47411575	-6.8170763	3.23997277	-2.1040536	0.03537377	0.99840724
Ak8	20.2872668	-2.1223686	1.00964753	-2.1020886	0.03554552	0.99840724
Inpp4a	1399.67693	-0.4040271	0.19239562	-2.0999805	0.03573055	0.99840724
Gm37485	7.14257316	-4.873362	2.32076425	-2.0998953	0.03573805	0.99840724
Gm6566	19.0333811	-2.2761217	1.08456661	-2.0986463	0.03584809	0.99840724
Cradd	156.016256	-0.6958656	0.33165941	-2.0981331	0.03589339	0.99840724
Gm27042	40.2008896	-1.9574557	0.93371447	-2.0964178	0.03604514	0.99840724
Gm2986	10.1475881	-3.3260108	1.58711545	-2.0956326	0.0361148	0.99840724
Etohd2	85.3414263	-1.0429724	0.49829607	-2.0930777	0.03634222	0.99840724
Man2a2	438.028322	-0.7155377	0.34186663	-2.0930317	0.03634632	0.99840724
Numa1	2555.57233	-0.4677172	0.22348063	-2.092876	0.03636023	0.99840724
Hba-ps4	5.06176626	-6.0735693	2.90360249	-2.0917358	0.03646215	0.99840724
Nxn	48.6318311	-1.457382	0.69879238	-2.0855723	0.03701739	0.99840724
Adcy10	14.1431539	-4.7149531	2.2619584	-2.0844561	0.0371187	0.99840724
Rccd1	213.153348	-0.7392988	0.35492311	-2.0829829	0.03725278	0.99840724
C530050E15Rik	260.273416	-1.1525187	0.55342377	-2.0825248	0.03729456	0.99840724
Gm10033	187.257659	-0.7449673	0.35774669	-2.0823877	0.03730707	0.99840724
Gm48207	8.25111271	-6.7785862	3.25782873	-2.0807067	0.03746076	0.99840724
Plp1	39.9654944	-2.0199578	0.97202757	-2.078087	0.03770135	0.99840724

Gm43162	48.0608702	-1.2399327	0.59734688	-2.0757331	0.03791865	0.99840724
Gm31834	12.6861814	-3.7585122	1.81160955	-2.0746811	0.0380161	0.99840724
Slc4a11	14.4174773	-3.7832316	1.82428921	-2.0738113	0.03809684	0.99840724
Hpn	87.9799022	-1.1022556	0.53182939	-2.0725738	0.03821197	0.99840724
Gm15859	24.0778445	-2.1810827	1.05287553	-2.0715484	0.03830757	0.99840724
Lin52	206.23745	-0.5884592	0.28412865	-2.0711014	0.03834932	0.99840724
Ppic	209.642862	-1.2130318	0.58845401	-2.0613876	0.03926608	0.99840724
Zfp791	17.2932937	-4.0054813	1.94528061	-2.0590763	0.03948693	0.99840724
Glt28d2	38.682685	-1.2211645	0.59313151	-2.0588427	0.03950931	0.99840724
Kcna2	77.008628	-1.2943918	0.63069689	-2.0523199	0.04013858	0.99840724
Tshz1	288.986226	-0.7772137	0.37878903	-2.0518379	0.04018542	0.99840724
D130043K22Rik	25.5965698	-2.0255924	0.9881391	-2.0499062	0.04037359	0.99840724
Trip11	1077.63484	-0.5266711	0.25704422	-2.0489514	0.04046687	0.99840724
Zfp595	218.31319	-0.9666901	0.47200866	-2.0480346	0.04055661	0.99840724
Tet3	1354.69031	-0.5124171	0.25042154	-2.046218	0.04073492	0.99840724
St8sia4	1127.58349	-0.7227001	0.35320703	-2.046109	0.04074564	0.99840724
Gm43024	32.6518232	-2.7287375	1.33549538	-2.04324	0.04102869	0.99840724
Pogk	216.663015	-0.7351137	0.35996819	-2.0421629	0.04113538	0.99840724
Zfp862-ps	228.024588	-0.9349232	0.45826661	-2.0401295	0.04133743	0.99840724
Abca1	160.473329	-0.7434567	0.3646636	-2.0387468	0.04147531	0.99840724
Gm43328	53.7552415	-1.4715312	0.72349637	-2.0339164	0.04196002	0.99840724
Sbk1	227.920392	-0.6004437	0.29523048	-2.0338134	0.04197041	0.99840724
Ighv7-4	8.69014321	-5.8684767	2.88653381	-2.0330532	0.04204715	0.99840724
Acad11	204.295017	-0.8344835	0.41048567	-2.0329175	0.04206086	0.99840724

Mrgprb1	7.80510662	-6.6983809	3.29598749	-2.0322835	0.04212497	0.99840724
Gm29480	7.80510662	-6.6983809	3.29598749	-2.0322835	0.04212497	0.99840724
Gm17816	49.1161193	-1.1099778	0.54692521	-2.0294873	0.04240868	0.99840724
Kifc2	170.680661	-0.7761121	0.3829216	-2.0268171	0.04268112	0.99840724
Apc	571.411415	-0.8023632	0.39627447	-2.0247663	0.04289137	0.99840724
Gm19967	19.1046379	-1.7964789	0.88732945	-2.0245907	0.04290941	0.99840724
Gm42659	203.28905	-0.6738463	0.3341183	-2.0167895	0.04371748	0.99840724
Ptprk	43.630238	-1.8595242	0.92234637	-2.0160801	0.0437916	0.99840724
4833421G17Rik	169.660502	-1.0499248	0.52178357	-2.0121845	0.04420049	0.99840724
Pax9	7.58210357	-6.6565428	3.31641539	-2.0071499	0.0447337	0.99840724
4930563J15Rik	7.58210357	-6.6565428	3.31641539	-2.0071499	0.0447337	0.99840724
Tab1	663.595576	-0.5326745	0.26557519	-2.0057389	0.04488412	0.99840724
Papd7	149.121718	-1.0243049	0.5111792	-2.0038078	0.04509065	0.99840724
Klf12	24.3335318	-2.0723456	1.03559296	-2.0011198	0.04537948	0.99840724
Mpg	243.214605	-0.5055133	0.25284027	-1.9993385	0.04557174	0.99840724
Zfyve26	524.658544	-0.5791666	0.28998781	-1.9972103	0.04580234	0.99840724
BC043934	223.870429	-0.7153476	0.35819105	-1.9971119	0.04581303	0.99840724
AC163354.1	36.2729504	-1.6590883	0.83123249	-1.9959377	0.0459407	0.99840724
Gm37215	52.758322	-1.6193867	0.8124306	-1.9932615	0.04623281	0.99840724
Exph5	54.5353593	-1.227522	0.61685975	-1.9899531	0.0465961	0.99840724
Tnk2	242.194511	-0.5316413	0.2671708	-1.9898928	0.04660275	0.99840724
Trav17	44.6907377	-1.0225061	0.51424373	-1.9883687	0.04677093	0.99840724
Gm6855	6.05936361	-4.5360418	2.28183696	-1.9878904	0.04682381	0.99840724
Dnajc12	56.4342	-1.3952967	0.70337884	-1.9837058	0.04728865	0.99840724

Eme2	204.46877	-0.7193952	0.36276221	-1.9831041	0.0473558	0.99840724
Khdc1a	11.62864	-3.9737212	2.00532531	-1.9815843	0.04752578	0.99840724
A430078I02Rik	11.1939951	-2.7905265	1.40938667	-1.9799581	0.04770824	0.99840724
Sh3d19	60.1898424	-1.1168044	0.5642235	-1.9793652	0.0477749	0.99840724
Slc25a36	779.03037	-0.6088301	0.30761453	-1.9791981	0.0477937	0.99840724
Gm38228	112.513279	-1.3124291	0.66332855	-1.9785506	0.04786663	0.99840724
Tecta	6.73440572	-4.7891268	2.4225142	-1.9769241	0.0480502	0.99840724
Zfp597	147.023233	-1.0816389	0.54803035	-1.9736843	0.04841765	0.99840724
Xkr6	15.0734578	2.58319894	1.30919915	1.97311382	0.0484826	0.99840724
Phf3	646.079503	-0.5954957	0.30181475	-1.9730502	0.04848984	0.99840724
Trpm4	181.864705	-0.8314135	0.4214671	-1.9726651	0.04853373	0.99840724
Zfp78	60.4723985	-0.9710055	0.49258886	-1.9712291	0.04869767	0.99840724
2010007H06Rik	41.9167909	-1.6222257	0.8244381	-1.9676744	0.04910551	0.99840724
4732496C06Rik	55.7164958	-1.1515738	0.58534528	-1.9673411	0.0491439	0.99840724
Ddx19b	364.925468	-0.4996168	0.2541129	-1.9661214	0.04928459	0.99840724
Usp36	558.910318	-0.3778656	0.19221473	-1.9658514	0.04931578	0.99840724
Gm17477	22.4453728	-1.5290906	0.77801948	-1.9653628	0.04937226	0.99840724
Wwox	122.438918	-0.7247985	0.36889395	-1.9647884	0.04943873	0.99840724
Atm	428.245146	-0.828564	0.4224641	-1.9612648	0.04984814	0.99840724
Gm38403	12.5925973	-4.4644329	2.27714241	-1.9605418	0.0499325	0.99840724
Gm29156	5.66779756	-5.2247395	2.66515521	-1.9603885	0.0499504	0.99840724
